# Behavioural analysis of multi-year satellite telemetry data provides insight into narwhal (*Monodon monoceros*) winter prey selection in Baffin Bay

**DOI:** 10.1371/journal.pone.0330928

**Published:** 2025-09-02

**Authors:** Claire A. Hornby, Ron R. Togunov, Brett T. McClintock, Cortney A. Watt

**Affiliations:** 1 Fisheries and Oceans Canada (DFO), Freshwater Institute, Winnipeg, Manitoba, Canada; 2 Norwegian University of Science and Technology (NTNU), Trondheim, Norway; 3 Marine Mammal Laboratory, NOAA-NMFS Alaska Fisheries Science Center, Seattle, Washington, United States of America; 4 Department of Biological Sciences, University of Manitoba, Winnipeg, Manitoba, Canada; Kobe University, JAPAN

## Abstract

Narwhals (*Monodon monoceros*) are deep-diving Arctic cetaceans that migrate seasonally between summering and wintering grounds. The Baffin Bay population overwinters in southern Baffin Bay and Davis Strait, where they are known to forage on high-energy benthic prey. Studying narwhal winter behaviour and prey preference has been challenged by their remote distribution and limited lifespan of satellite tags deployed in summer, restricting data on their habitat use and foraging strategies. Since prey consumption is thought to peak in the winter, understanding narwhal diet plasticity in a rapidly changing environment like Baffin Bay is critical. This study developed unique methods to examine four years of irregular satellite telemetry data from 22 narwhals tagged in their summering grounds. Locations and recorded diving data from the overwintering area were isolated, and a hidden Markov model was used to define three behaviours (“surface”, “pelagic”, and “deep-water” diving). We further examined the effects of five covariates on these behaviours to provide insight into the spatial patterns of narwhal winter prey preference. Narwhal behaviours were dominated by diving, with 37% of their time spent in pelagic waters and 40% in deep-water, while only 22% of their time was spent in surface related behaviours. Deep-water behaviours increased later in the day and into the winter season and occurred frequently in the center trough of Baffin Bay before (66°- 69°) and across Davis Strait (65° - 67°). In contrast, pelagic behaviours declined as the winter season progressed and occurred earlier in the day. Narwhals occupying the northern overwintering area exhibited more pelagic behaviours, despite it being deeper, suggesting different foraging strategies across their winter range. Our study identified behaviours suggestive of a variable winter diet and provided insight on the spatial nature of these behaviours across the winter season. The methods developed in this study present new opportunities for analysing lower resolution satellite tracking data. With advancements in bio-logging technology and remote field methods, the ability to successfully document changes in winter space use and fine-scale foraging behaviours may be possible for narwhal in the future.

## Introduction

Narwhal (*Monodon monoceros*) exploit remote ice-associated habitats in the Arctic, and their wide-ranging seasonal habitat use make them challenging to study year-round. This medium-sized cetacean is elusive, can dive deep (>1500 meters), and exhibits site fidelity to the many isolated fiords, bays, and inlets across the Canadian high Arctic and northwest Greenland [1,2]. To date, much of our knowledge on narwhal migration, distribution, diving ability, and habitat use has come from telemetry studies, and the use of fixed satellite-linked time-depth recorders to collect information on horizontal movement and diving behaviour across space and time [3–9]. Specifically, satellite tracking has been one of the integral methods for gaining insight into the winter behaviour of narwhals in remote offshore areas such as southern Baffin Bay (hereafter BB) [3,8,10,11]. Despite this, documenting and advancing our knowledge of seasonal changes in narwhal habitat selection and foraging behaviour is often hindered by data collection challenges related to satellite tag technology and longevity, for example tags will often periodically go offline, collect intermittent data (i.e., pre-programmed duty cycles to save battery), or completely going offline before the animals reaches the winter area, resulting in sparse datasets [12]. Fortunately, diet studies using stomach content analysis, and stable isotope and fatty acid analysis of tissues from hunted narwhals have helped to provide a more comprehensive understanding of narwhal prey preferences in different populations and seasons [6,8,9,13,14].

In Canada, multi-annual tagging projects focused on the BB narwhal population date back to 1997 [4,10,15], with research questions primarily driven by data collected in the summering grounds of Admiralty Inlet (AI) and Eclipse Sound (ES) (June-September), where narwhal aggregate close to shore and are harvested for subsistence by communities on Baffin Island and Greenland [16]. By late fall (October-November), these whales come together during migration and travel approximately 1700 km to two core offshore wintering grounds in BB, one area more central, west of the continental slope, between 69°30’N and 70°30’N at 63°W [1,2,4], and another in southern BB and northern Davis Strait (the shallow area between BB and the Labrador Sea) along the continental slope between 69°30’N and 68°N at 58’W [3,5,15]. Here they reside in the dense offshore pack ice (at times >95% ice cover) or along the fast ice edge of the continental shelf [3,15,17–19] for the remainder of the winter. Northward migration back to summering areas begins as the ice breaks in spring (April-May) [4,10,20].

Likely driven by resource availability, BB narwhals display unique foraging strategies across their range, often using the deepest part of the water column [3,5,21], and increasing diving depths as the winter season progresses [6,9,11,21]. Specifically, narwhals residing in the overwintering area tend to prefer deep offshore habitat [11], often spend a significant amount of time in 75%–100% of the total bathymetric depth [9], and bottom temperature ranges that suggest a preference for benthic prey [5,22]. Narwhals can successfully feed at a variety of depths, but have typically been described as dietary specialists with little behavioural flexibility [9,23]. In the winter, it is generally believed that the most important prey for narwhal in this region is Greenland halibut (*Reinhardtius hippoglossoides*) [5,6,24]; a benthic dweller, most common between 400 and 1500 m [25–27]. However, diet studies using stomach contents have also identified important pelagic prey such as squid (*Gonatus fabricii*), Arctic cod (*Boregadus saida*), Polar cod (*Arctogadus glacialis*), and capelin (*Mallotus villosus*) [6]. The majority of stomach contents have been collected from narwhals hunted during the summer and occasionally spring at the floe edge. However, Laidre and Heide-Jørgensen [6] investigated stomachs from harvested whales taken off west Greenland and found they contained considerably more, and fresher, food during the winter than stomachs sampled in the summer. Although stomach contents can reveal the diversity of prey consumed, they can only provide information on the most recent meal (i.e., not what was assimilated into the tissues); therefore, the mismatch in collection times adds some limitations in their ability to accurately describe preference for prey items available across their winter range [23,28]. Interestingly, Watt et al. [23] examined diet signatures in BB narwhal tissues, which are more reflective of trophic level and spatial foraging distribution, and observed that Greenland halibut was not the primary prey type, noting high proportions (>50% of their diet) of shrimp (*Pandalus borealis*), and Arctic and Polar cod. Although tissue turn over rates may be a limiting factor in these results (see [23] for details), the inconsistent findings between diving and diet studies do suggest that BB narwhal may have a more variable winter diet than previously thought.

A better understanding of diet plasticity in a rapidly changing environment like BB (e.g., see [29]) is critical, as narwhals have been assessed as one the most sensitive of Arctic endemic marine mammals to climate change [30]. Further, their capacity to adjust long-term adaptations, like fixed migratory patterns and prey preferences to climate-induced perturbations remains relatively unknown, and an important consideration for population dynamics [12,31,32]. To deepen our understanding of BB narwhal behaviour within their overwintering grounds, we analysed movement data collected by satellite tracking devices deployed each summer from 2009 to 2012. Specifically, we modeled narwhal behaviour in relation to habitat covariates using behavioural metrics derived from horizontal (i.e., latitude and longitude) and vertical (i.e., dive) data, allowing us to distinguish between behaviours indicative of foraging on deep-benthic versus other pelagic prey. For a species like narwhal that exist in 3-dimensional space, the use of auxiliary information, such as diving depths and time spent at depth, is fundamental for understanding habitat utilization [33]. Predicted habitat associations, as well as empirically predicted behavioural states, were evaluated to identify and differentiate the spatial structure and distribution of pelagic and foraging dives in the overwintering area, including dives to the benthic environment. The methods developed in this study have important applications for utilizing irregular satellite tracking data that may not be possible with standard movement modeling analyses.

## Materials and methods

### Study area and field tagging

**Fig 1:**
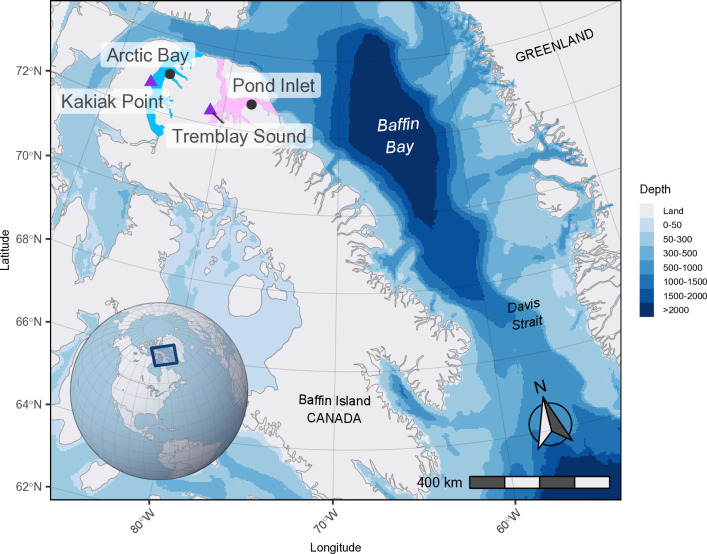
Map of the Baffin Bay-Davis Strait study area, situated between Canada and Greenland. The Baffin Bay narwhal population summering areas of Admiralty Inlet (blue) and Eclipse Sound (pink) are shown, along with the two field tagging sites at Kakiak Point (2009) and Tremblay Sound (2010-2012). Bathymetry raster (4000 m by 4000 m) from the International Bathymetric Chart of the Arctic Ocean, IBCAO, dataset version 4.3 [34].

Narwhals (24) were captured and tagged at Kakiak Point (72º 41’ 00” N, 86º 41’ 20” W) in Admiralty Inlet near the community of Arctic Bay, Nunavut, in August 2009, and in Tremblay Sound (72º 21’ 23” N, 81º 6’ 24” W) near the community of Pond Inlet, Nunavut, in August 2010, 2011, and 2012 (Fig 1). Details pertaining to the wild capture of narwhals have been described previously in Orr et al. [35], Dietz et al. [10,15], and Heide-Jørgensen et al. [4,36]. All captured narwhals were equipped with an embedded SPLASH tag (Wildlife Computers, Redmond, WA, USA) and in-depth details from the 2009−2012 tagging program can be found in Watt et al. [8,9], Kenyon et al. [11], and Shuert et al. [7,12]. Permission for capturing, handing, and tagging of narwhals was provided by Fisheries and Oceans Canada (DFO), and the communities of Arctic Bay and Pond Inlet. Animal handing procedures were reviewed each year and approved by the DFO Freshwater Institute Animal Care Committee (FWI-ACC-2009–024, FWI-ACC-2010–001, FWI-ACC-2011–016, and FWI-ACC-2012–009), and a License to Fish for Scientific Purposes granted (license numbers S-09/10–1015-NU, S-10/11–1029-NU, and S-11/12/1039-NU).

### Satellite telemetry data

The Argos system (CLS America) was used to receive transmissions and to assign each whale an ID, or PTT (platform terminal transmitters). Transmissions from the tags were programmed to begin from the time the tag was equipped on the animal (Table 1), and transmission times were set to collect higher resolution data in the summering area (transmissions daily June-September 30) and lower resolution data during the migration and overwintering period to save battery life, known as a duty cycle (transmissions every 4 days in 2009 and 3 days in 2010–12, from October 1-May 31). Satellite locations and location quality classes (originally provided by a least-squares algorithm in 2009–2012) were re-analysed using an improved Kalman filtering (KF) algorithm [37,38], which provided estimates of location and measurement error ellipses oriented around each point [39].

**Table 1:** Metadata for narwhals (N = 22) satellite tagged in Admiralty Inlet (AI) in 2009 and Eclipse Sound (ES) 2010-2012 that reached the overwintering area in southern Baffin Bay. The first and last date (yyyy-mm-dd) in the defined overwintering area were estimated using time-series plots, resulting in the number of winter days/locations for each ID used in the hidden Markov model (HMM) analysis.

ID	Tagging location	Sex	First transmission	Last transmission	First date in winter area	Last date in winter area	N. winter days	N. winter locations
39309	AI	M	2009-08-16	2010-05-02	2009-11-07	2010-05-02	175	163
39313	AI	F	2009-08-16	2010-02-11	2009-11-07	2010-02-11	96	135
39287	AI	M	2009-08-17	2010-06-11	2009-11-07	2010-04-20	163	218
39311	AI	M	2009-08-17	2010-06-03	2009-11-23	2010-03-27	123	235
39249	AI	F	2009-08-19	2010-01-10	2009-11-11	2010-01-10	60	81
39256	AI	M	2009-08-19	2010-07-05	2009-11-07	2010-04-24	167	333
51871	ES	M	2010-08-21	2011-04-28	2010-10-16	2011-04-28	194	447
51872	ES	M	2010-08-21	2011-06-08	2010-10-22	2011-05-22	212	481
51873	ES	F	2010-08-22	2011-07-31	2010-11-13	2011-05-10	177	591
51874	ES	F	2010-08-22	2011-02-26	2010-11-01	2011-02-26	117	95
51875	ES	F	2010-08-24	2011-01-25	2010-11-07	2011-01-25	79	54
51876	ES	F	2011-08-16	2012-02-13	2011-11-07	2012-02-13	98	376
51878	ES	M	2011-08-16	2011-12-22	2011-11-06	2011-12-22	46	87
51879	ES	F	2011-08-17	2012-06-26	2011-11-10	2012-04-07	148	599
39315	ES	F	2011-08-19	2011-12-22	2011-11-16	2011-12-22	36	86
39270	ES	F	2011-08-20	2012-03-07	2011-11-07	2012-03-07	121	259
39314	ES	F	2011-08-20	2012-03-31	2011-11-07	2012-03-31	144	413
57590	ES	F	2011-08-20	2012-06-16	2011-11-04	2012-05-02	180	462
115957	ES	F	2012-08-14	2012-12-19	2012-10-31	2012-12-19	49	158
115958	ES	M	2012-08-17	2012-12-22	2012-11-10	2012-12-22	42	103
115959	ES	F	2012-08-18	2012-12-19	2012-11-16	2012-12-19	33	54
115960	ES	F	2012-08-19	2012-12-16	2012-11-13	2012-12-16	33	60

The tags also collected binned dive data throughout the deployment. In all years, dive data were sampled every 10 seconds, and then grouped into depth bins summarized every 6 h directly prior to the transmission (always starting daily on the hour at 03:00 GMT). The depth reading to determine the start and end of a dive was set at 4 m. The dive bin programming for the 2009 and 2010–2012 tags have some inconsistencies, mainly the 2010–2012 tags collected deeper depth bins (up to 1800 m) (dive bins shown in S1 Table in S1 File). Two main dive metrics were used in this study: (1) the Dive Maximum Depth (DMD) record, which represents the number of dives (frequency) to a maximum depth within the pre-programmed depth bin range, and (2) Time at Depth (TAD), which represents the proportion of time (in seconds) spent in the specified depth bin range (shown as a percentage of the total 6 h collection period).

**Fig 2:**
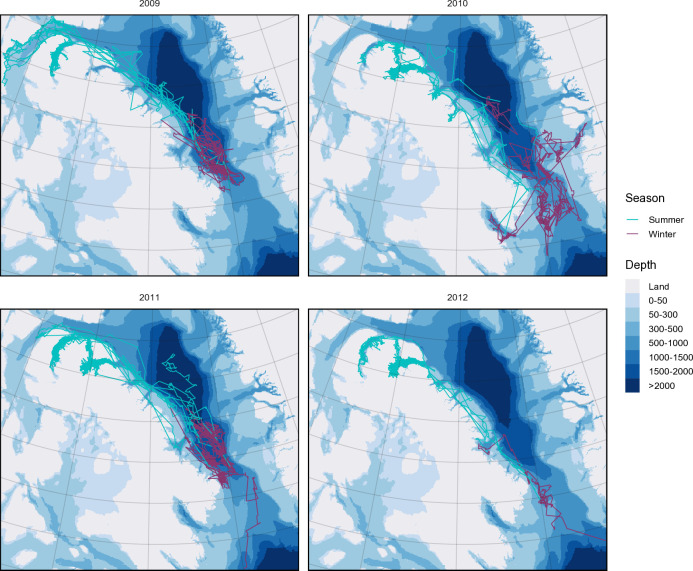
Argos locations and tracks of Baffin Bay narwhals (N = 22) tagged in 2009 in Admiralty Inlet and 2010–2012 in Eclipse Sound that reached the overwintering area in southern Baffin Bay. Tracks are split into the defined summer (turquoise) and winter (purple) seasons. Bathymetry raster (4000 m by 4000 m) from the International Bathymetric Chart of the Arctic Ocean, IBCAO, dataset version 4.3 [34].

All narwhal telemetry data were analysed in R version 4.3.3 [40]. Of the 24 narwhals tagged from 2009–2012, 22 of them reached the defined overwintering ground in southern BB (details on the deployments of all tagged whales can be found in [1,8,9,11]) (Table 1, Fig 2). Locations from these whales (spanning the whole deployment from summer to winter) (Table 1) were first processed using data cleaning practices common for telemetry data, such as removing any duplicates and spatial outliers (i.e., obviously wrong locations many kilometers away or on land) from the study area using the R package argosfilter [41], and a course speed filter of 10 m/sec, which is more than double the biological vertical maximum estimated for this species [14] and similar to other movement studies [42]. There tends to be a visible signal in the speed and transiting behaviour of narwhals as they migrate south and reach the overwintering area [12]. As such, winter locations for each individual (Fig 2) were defined by manually identifying the first and last location in their winter range using time-series plots of standardized values of surface time, and x and y coordinates (similar to [10,11]) (see S1 Fig for example).

### Predicting temporally-regular locations

We used hidden Markov models (HMM) to investigate the relationship of different behavioural states, and the remaining data manipulation and analysis of the 2009–2012 narwhal locations and dive data were completed using the R package momentuHMM [43], with unique adjustments made to handle large data gaps. As mentioned previously, the Argos locational data are subject to measurement error and were not collected at equally spaced temporal periods, also known as temporal irregularity. Due to duty cycling and other unknown factors, such as environmental conditions (i.e., heavy ice), tag placement, and/or behaviour (i.e., deep dives), there are certain periods throughout the deployments when the tags do not transmit for multiple days (S2 Fig). To predict missing locations and account for location error along each track, a continuous-time correlated random walk (CTCRW) model [44], was first fitted to the whole duration (summer and winter) of the tag deployment using the crawlWrap function. Having sizable gaps in tag transmissions can create problems with the accuracy and success of predictions in the CTCRW model (i.e., long stretches of no data will often be observed as predicted points interpolated as a straight line); therefore, the tracks were segmented into periods of consistent observed locations, when there were >7 days without a location or dive record (see S1 Appendix in S1 File).

### Data streams and environmental covariates

To aid in predicting behavioural foraging states and distinguishing between pelagic and deep-water environments targeted by narwhals, various data streams were initially tested for autocorrelation and selected accordingly (see S2 Appendix in S1 File and S3-S6 Figs). For movement, we selected mean speed (m hr^-1^) and tortuosity (the ratio between three-step path length L and straight-line distance D between the first and last locations) at time *t*. In order to model tortuosity (can be any value ≥ 1) using a gamma distribution, which has a range > 0, we subtracted 1 from tortuosity:


$$tortuosity_{t}\;=\;\frac{L_{t,t+1}+L_{t+1,t+2}+L_{t+2,t+3}}{D_{t,t+3}}-1$$

The dive data streams selected were mean dive depth, relative dive depth (dive depth relative to the sea floor), time at surface (<6m, defined as not diving), and time at depth (>400 m).

Behavioural state transition probabilities were also modeled against three environmental covariates, distance to shore (shore), bathymetry (bath), and seafloor slope (slope), as they have all have been shown to impact narwhal winter habitat use, movement, and diving behaviour [9,11,15,42], along with location of forage fish species within the southern BB ecosystem. The maximum bath values were extracted from the gridded (200 m x 200 m resolution) International Bathymetric Chart of the Arctic Ocean (IBCAO) [34] and slope from the General Bathymetric Chart of the Oceans (GEBCO) [45]. Since individuals may travel significant distances during the 6-hour window represented by the dive bin data, bath was defined as the maximum value available within a 20 km buffer around the predicted locations, and slope was defined as the mean slope within 20 km. Distance to shore was derived based on distance to bathymetry of 0 m. In addition to these environmental factors, local time of day (hour) (i.e., UTC-4; 24-hour cosinor model [43]), and day of winter (wday; defined as days since September 1) were also investigated as possible predictors for the state transition probabilities.

### Model development and behaviour analysis

We developed a 3-state HMM intended to represent periods of predominantly surface activity (6–200 m; resting and/or travelling), pelagic diving within the water column (200–500 m), and deep water diving (>500 m). Due to size and complexity of the HMM, we used a step-wise approach integrating data streams, covariates, and covariate effects on emission and state transition probabilities (see S3 Appendix in S1 File).

In the context of HMMs, emission probabilities define the likelihood of observing each data stream value given a particular hidden state, thereby linking observable metrics (e.g., mean speed or dive depth) to these behavioural states. We modeled these probabilities using respective distributions: gamma for mean speed (Ms), tortuosity (Tr), surface time (Sf), and time at depth (Dp), and Weibull for mean depth (Md) and relative dive depth (Rd). Gamma distributions were defined by mean (μ) and standard deviation (σ) parameters, while the Weibull distribution is defined by a shape (κ) and scale (λ) parameters:


$$O_{S,t}\sim {{\{}_{gamma\left(\mu_{S,t}^{\left(O\right)},\sigma_{S,t}^{\left(O\right)}\right)}^{weibull\left(\kappa_{S,t}^{\left(O\right)},\lambda_{S,t}^{\left(O\right)}\right)}}_{otherwise,}^{if\;O\in \left\{Md,Rd\right\}}$$

where *O* is the observed data stream, and *S* is the state at time *t*. Time at depth, Dp, had an additional zero-mass parameter (z) estimated because some periods had no records (i.e., whales did not dive) in deep water (i.e., Dp = 0); however, the gamma distribution only supports values >0.

Three key covariate effects on emission probabilities were applied to relevant diving data-streams and states. First, an effect of bathymetry was applied to the distributions of mean depth, relative dive depth, surface time, and deep time, for only the diving states. The rationale for this being that available depths associated with pelagic and deep waters vary with bathymetry, setting a limit on narwhal dive depth, and influences the characteristics of dive data streams and states. For example, benthic dives in shallow regions, like over the continental shelf, will have shallower mean depths compared to deeper waters. Similarly, we might assume diving animals need to rest longer following more energetic deep dives, and surface times following benthic dives may be longer in deeper waters. For the second covariate effect, surface time was modeled as a function of winter day. This was used to capture behaviours related to narwhals shifting from migration (typically defined by traveling and higher surface time) to winter foraging behaviours as dive frequency increases. With higher-resolution data, winter date could be included as a covariate affecting state-transition probabilities. However, given the coarser 6-hour resolution (i.e., dive data averages multiple dives), there are likely a number of internal state transitions within this time frame or fine-scale states that only occur during migration (i.e., prolonged directional travel). This could result in changing the state-specific distribution (i.e., state switching) of all dive data streams when whales spend more time at the surface.

Lastly, the effect of tag programming was important since tags deployed in 2009 had different depth bins than in 2010–2012. Since the tag programming impacts the apparent mean depth, relative dive depth, and time at depth, we modelled these data streams as functions of tag programming, with the 2010–2012 programming serving as the reference. Specifically, the tag programming acted as an intercept term for the shape and scale parameters of the mean depth, relative dive depth, and time at depth data streams for the shared states that would be affected by the difference (see S3 Appendix in S1 File and S7 Fig).

In all covariate effects on emission distribution, the same formula was used to affect both parameters of the given distribution (i.e., μ and σ of the gamma distribution and κ and λ of the Weibull distributions). For example, the following are the definitions of the shape, κ, and scale, λ, parameters of the mean depth distribution showing the state-specific effect of bathymetry on parameters (i.e., on states 2 and 3 but not on state 1) (formulas for relative dive depth, surface time, and time at depth are shown in S3 Appendix in S1 File):


$$\kappa_{S,t}^{\left(Md\right)}={{\{}_{\beta_{0,S}^{\left(Md\right)}+\beta_{1,S}^{\left(Md\right)}\;prog+\beta_{2,S}^{\left(Md\right)}\;bath}^{\beta_{0,S}^{\left(Md\right)}+\beta_{1,S}^{\left(Md\right)}\;prog}}_{Otherwise}^{if\;S=1}$$

$$\lambda_{S,t}^{\left(Md\right)}={{\{}_{\beta_{3,S}^{\left(Md\right)}+\beta_{4,S}^{\left(Md\right)}\;prog+\beta_{5,S}^{\left(Md\right)}\;bath}^{\beta_{3,S}^{\left(Md\right)}+\beta_{4,S}^{\left(Md\right)}\;prog}}_{Otherwise}^{if\;S=1}$$

Finally, to examine the spatial distribution of behavioural states in our study subjects, we implemented a structured approach by first determining the most likely sequence of states using the Viterbi algorithm [46]. These decoded states were rasterised to a 500 m grid. Given that some states are generally more common than others, simply quantifying which state was most frequent would only highlight common states (in each cell) and may mask variation among underlying states. Following Togunov et al. [47], we identified which cell was disproportionately most frequent within each cell relative to its occurrence across the overwintering area. Specifically, for a given cell i, we identified the state 𝐒′ as the one that exhibits the highest within-cell frequency relative to its global frequency following:


S′i=(argmaxS=1,2,3,4) (Ni(S)NiN(S)N),

where $N_{i}$ is the total number of steps in cell i across all states, $N_{i}^{\left(S\right)}$ is the number of steps in cell i for state S, N is the total number of steps across all cells and states, and $N^{\left(S\right)}$ is the number of steps across all cells for state S. This methodological refinement ensures a more balanced and accurate representation of state distribution, highlighting areas of disproportionate state activity without the skew introduced by overall state frequency.

## Results

Satellite tags deployed on 22 narwhals overwintering in southern BB (2009–2012) transmitted for 33–212 days (Table 1). The majority of tags lasted into the winter months, with 64% transmitting at least to the end of January and 36% to the end of March. The earliest recorded arrival to the wintering area was October 16, while most whales arrived between late October and early November and departed between late March and May (Table 1). Narwhal tracks from 2009–2010 and 2011–2012 were more tightly grouped throughout southern BB in the winter, while movements from 2010–2011 were more extensive (Fig 2). Narwhals tagged in Tremblay Sound (2010–2012) moved farther south in the overwintering area, occupying the Davis Strait sill (south of 66° latitude), whereas the AI tagged whales from 2009 stayed further north (Fig 2). In 2010, two narwhals (51871 and 51872) entered Cumberland Sound in late October, and one of them (51872) later spent about a month in Disko Bay, Greenland (December–January). More detailed descriptions of individual narwhal movements can be found in [11,20]. Narwhals had access to a wide range of bathymetry throughout their winter range, with varied depths across Davis Strait, from 500 to 900 m; however, much of the overwintering area is over deep-water exceeding 1000 m. The weighted mean diving depth throughout the winter was 207 ± 99 m, with maximum dive depths exceeding 1400 m.

Raw Argos locations (N = 5490) were compiled for all 22 narwhals across four winter seasons. The largest dataset was from 2011 (773 winter days, 2282 locations), followed by 2010 (779 winter days, 1668 locations) and 2009 (784 winter days, 1165 locations). In 2012, all tags stopped transmitting in late December, yielding 157 winter days and 375 locations. After interpolating all the tracks (i.e., interpolating at 2-hour time steps and summarizing at 6-hour), the number of steps used for the HMM totaled 9107.

**Fig 3:**
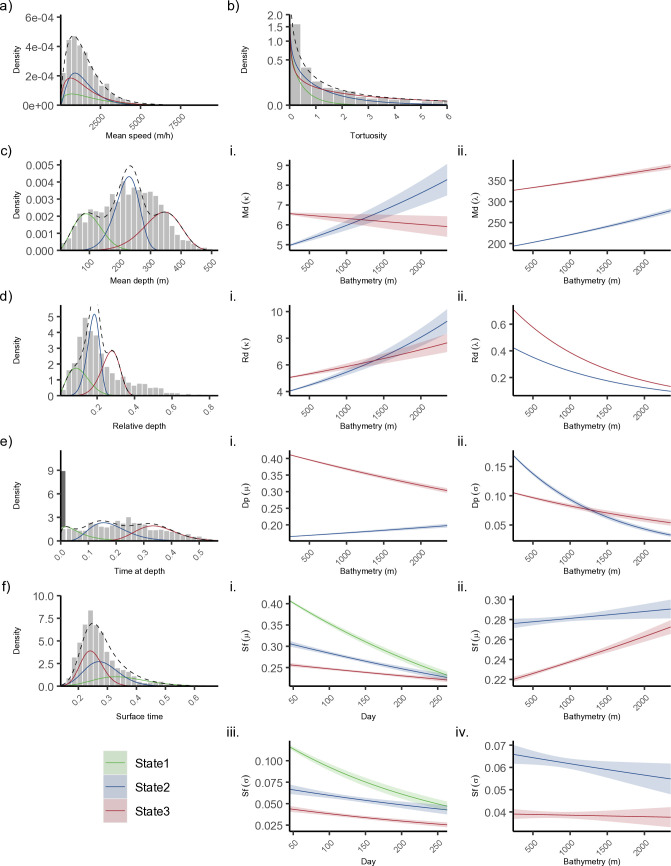
Hidden Markov model (HMM) histograms and decoded states (Viterbi) for three behaviours of overwintering Baffin Bay narwhals. States 1 (surface activity; green), 2 (pelagic diving; blue), and 3 (deep diving; red) are shown with respect to data streams: mean speed (Ms), tortuosity (Tr), surface time (Sf), mean depth (Md), relative dive depth (Rd), and time at depth (Dp). Covariate effects on the emission probabilities for shape (κ), scale (λ), mean (μ), and standard deviation (σ) included effect of available bathymetry on diving behaviour (Md, Rd, Sf, and Dd), effect of day of winter on surface time, and effect of tag programming on diving data streams. Covariate effects are numbered using Roman numerals and correspond to the histogram on the same row. Distributions for Md, Rd, and Dp, and Sf assume 2010 + tag programming, and a fixed mean bathymetry of 1379 m and winter day of 100. The first column of data for Dp highlighted in dark grey represents zeros that were modeled by the zero-mass parameter. Ribbons represent the estimated 95% confidence interval. Histograms exclude values of tags deployed in 2009.

Based on state-specific parameter estimates, state 1 (defined as no forage diving/surface behaviours) was characterized by higher mean speed (mean ± SD, 1902 ± 1512 m hr^-1^) (Fig 3a), with a maintained directional persistence toward a straight path and lower value of tortuosity (0.22 ± 0.26) (Fig 3b). In this state, the mean depths were shallow (< 100 m) (98 ± 43 m) (Fig 3c) and whales spent the highest proportion of time at the surface between all three states (32 ± 8% of time was spent < 6 m and 32% of the total steps stayed above 400 m) (Fig 3f). Of the remaining 68% of steps that reached 400 m, only 7 ± 6% of time was spent ≥ 400 m (i.e., 4 ± 4% of the overall time in state 1 was spent ≥ 400 m) (Fig 3d-e).

State 2 (defined as pelagic behaviours/diving) was characterized by moderate speeds (1456 ± 890 m) (Fig 3a) and intermediate tortuosity (0.5 ± 0.64) (Fig 3b). State 2 had moderate mean diving depths (220 ± 39 m) (Fig 3c) and whales spent less time at the surface than state 1 (27 ± 6%) (Fig 3f). A greater number of steps reached deep water (91% reaching ≥ 400 m), however, the mean portion of time spent below 400 m was only 17 ± 6% (Fig 3d-e).

State 3 (defined as deep-water behaviours/diving) included the lowest mean speed (154 ±  1185 m hr^-1^) (Fig 3a) and highest degree of tortuosity (1.0 ± 1.68) (Fig 3b). State 3 was characterized by the greatest mean depth (331 ± 62 m) (Fig 3c), with whales spending the least amount of time at the surface of all states (24 ± 4%) (Fig 3f) and the greatest amount of time > 400 m (around 100% of steps reaching ≥ 400 m, with a mean of 35 ± 7% time spent > 400 m) (Fig 3d-e).

Surface time was modeled as a function of winter day across all states and all three states showed a decline in surface mean time as winter date increased, with surface mean being the highest in state 1 early in the season, followed by state 2, and lowest in state 3 (Fig 3f(i)). Bathymetry effects were applied to states 2 and 3, and affected mean depth, relative dive depth, time at depth, and surface time data (Fig 3c-f). Mean diving depths were higher in state 3 at shallow depths and decreased as bathymetry increased (Fig 3c(i)). The relative diving depth was also higher in state 3 at shallow depths (Fig 3d(i)); however, the means of state 2 and 3 increased similarly at deeper depths. The mean time at depth parameter was higher in state 3 than state 2 and decreased with increasing depth (Fig 3e(i)). Mean surface time was higher in state 2 than state 3 at shallow to moderate depths (Fig 3f(ii)), while surface time increased more steeply in state 3 with increasing depth (Fig 3f(ii)).

**Fig 4:**
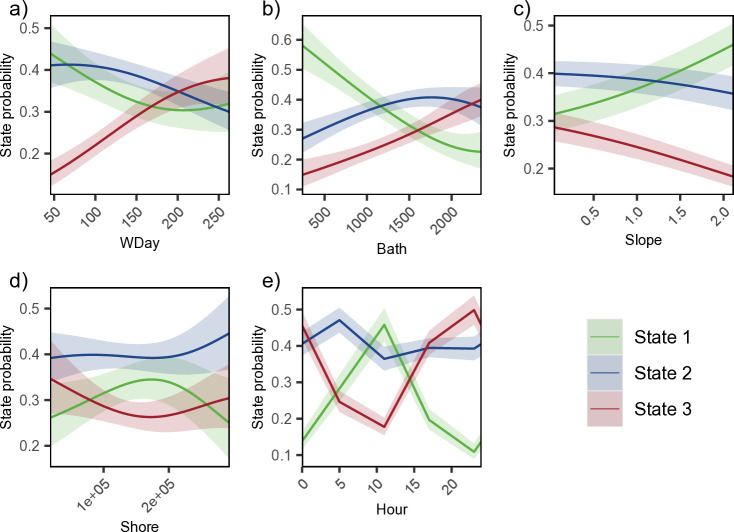
Stationary state probabilities of all behavioural states as functions of covariates. State 1 (green), 2 (blue), and 3 (red) shown as functions of winter day (wday), bathymetry (bath), seafloor slope (slope), distance to shore (shore) and local hour of day (hour; note x-axis is shown from 0 (midnight) to 23 hours). Shaded area represents the 95% confidence interval.

The stationary state probabilities as functions of the model covariates showed that the likelihood of state 1 and state 2 behaviours decreased as wday increased, whereas the probability of state 3 behaviours increased as wday increased (Fig 4a). State 1 likelihood decreased as bath increased and increased for state 2 and 3 (a continuous increase was observed in state 3, while state 2 increased and then leveled off at approximately 1500 m) (Fig 4b). For seafloor slope, state 2 behaviours increased as slope increased, with a peak observed at approximately 170 km from shore (Fig 4c-d). State 3 probability decreased with increasing slope and distances <200 km from shore (Fig 4c-d). A noticeable peak in state 1 behaviours were detected around noon before declining for the rest of the day (Fig 4e). While the reverse trend was observed for state 3, behaviours appeared to drop off in the morning and then steadily increase, peaking into the late afternoon and evening (Fig 4e). Slope, shore, and hour did not significantly impact behavioural state 2 (Fig 4c-e).

**Fig 5:**
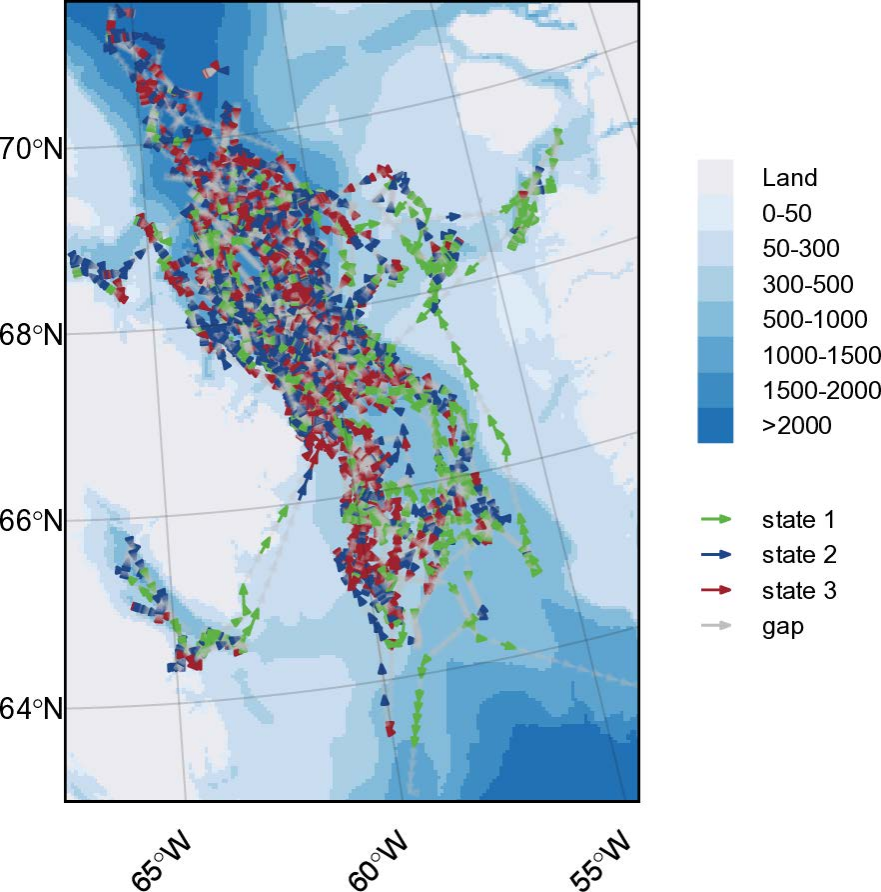
Map of decoded states for all 22 satellite tagged narwhals that reached southern Baffin Bay-Davis Strait. Tracks are coloured by decoded behavioural states (1 = green, 2 = blue, 3 = red) and data gaps are indicated in grey.

Based on the Viterbi-decoded states in relation to diving data streams, narwhals spent approximately 22% of their time in the overwintering area in behavioural state 1, 37% in state 2, and 40% in state 3. Combined decoded narwhal state behaviours across all years showed concentrated areas of state 2 and 3 were observed for all individuals in the central deep waters of BB extending all the way to Davis Strait (closer to Baffin Island than Greenland) (Fig 5). State 1 behaviours appeared to be dominated by narwhals 51871 and 51872, which as previously noted, had a significant amount of transiting/travel time to Cumberland Sound and Greenland in 2010.

**Fig 6:**
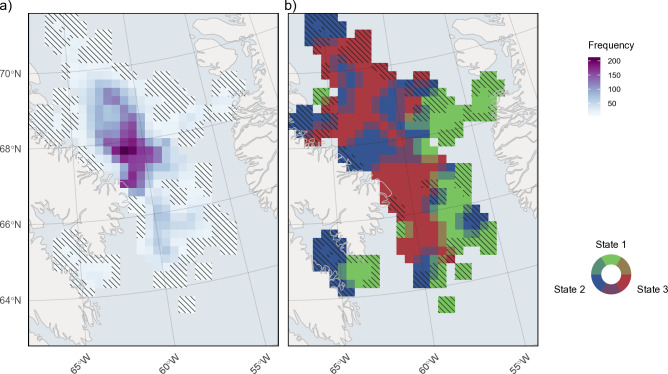
Winter distribution of decoded predicted states across all years. (a) Total number of steps that occurred in each cell (50 km by 50 km), which represents the total data that went into predictions for each cell (higher number of steps used indicates greater confidence in state predictions), and (b) calculated as the state ***S*** with the highest within-cell proportion relative to the frequency of each state across all cells. Cells with < 4 steps are not plotted, and hash lines represent cells with < 16 total steps, which indicate low certainty in those state predictions.

**Fig 7:**
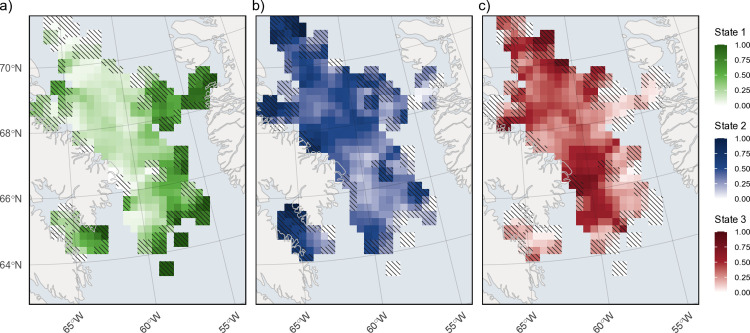
Predicted winter distribution of decoded behavioural states. (a) State 1 (green), (b) state 2 (blue), and (c) state 3 (red), dark areas represent high state frequency within each cell (50 km by 50 km). Cells with < 4 steps are not plotted, and hash lines represent cells with <16 total steps, which indicate low certainty in those state predictions.

**Fig 8:**
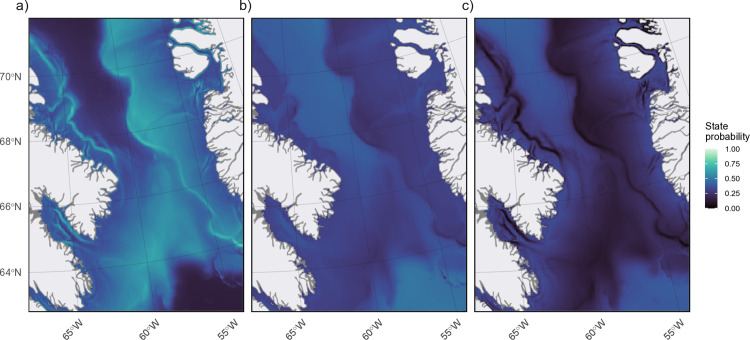
Map of predicted stationary state probabilities across narwhal overwintering area. All three states (panels a: state 1, b: state 2, c: state 3) mapped based on covariate (bathymetry, distance from shore, seafloor slope, winter day, and hour of day) values at each location.

The location data exhibits a non-uniform distribution across the overwintering area, with the highest concentration of locations around the southwest side of Baffin Island before Davis Strait (approximately 66°–69°) (Fig 6a). Relative to each state and proportion of locations across each cell, state 3 behaviours were more common closer to Baffin Island (68°–70°) and along the Davis Strait sill (65°–67°) (Figs 6b and 7c), while state 2 behaviours were more frequent north of 67° (Figs 6b and 7b). State 1 behaviours were concentrated along the shelf edges, particularly on the Greenland continental shelf (Figs 6b and 7a). Predicted stationary state probabilities mapped across the entire overwinter area to covariate values (Fig 8) further highlighted that state 1 probabilities increased along the shelf edges and in the southern overwintering region. Both state 2 and 3 behaviours have the highest probability in the deep-water basin of BB and state 2 is higher in the northern overwintering area. State 3 is nearly absent along the shelf edge and the central region south of Davis Strait (Fig 8).

**Fig 9:**
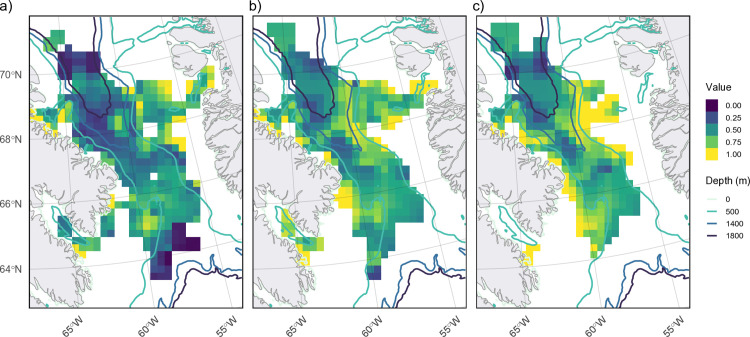
Maximum dive depth reached within each behavioural state. Maximum dive depth calculated as a proportion of available maximum depth for a) state 1, b) state 2, and c) state 3. Bathymetric contour lines (light to dark) indicate 0 m, 500 m, 1400 m, and 1800 m.

As expected, dives made to the bottom of the available bathymetry were the most common in states 2 and 3 (Fig 9), and narwhals reached at least 75% of the available bathymetry in 28% of steps. Maximum median diving depths in state 2 were not always to the bottom and more scattered across their range, but typically within 50–100% of the available bathymetry (Fig 9). Common benthic areas targeted by narwhals in state 2 included the northern part of Davis Strait, closer to southern Baffin Island (inside the 500 m isobath), and more north along the Greenland shelf, west of Disko Bay (appears to be along the shelf edge) (Fig 9). The maximum diving depths of narwhal in state 3 occurred primarily within 50–100% of the available bathymetry and occurred more frequently throughout the overwintering area (Fig 9). State 3 benthic diving behaviours appear to reach the seafloor in similar areas to state 2, but occur more in the southern part of the overwintering area and central deep basin of BB beyond 500 m.

## Discussion

Our study investigated narwhal telemetry data collected over four years from 2009–2012, representing one of the largest datasets to transmit into the winter season for the BB narwhal population. The locations and dive metrics from these 22 individuals were summarised into six movement anddive data streams, and used to characterise behavioural states indicative of different foraging habitats throughout the winter season. Given the temporal-resolution of this analysis, we selected a simpler 3-state HMM with the states representing periods composed of predominantly surface, pelagic, or deep behaviours (states 1–3, respectively). The surface state was characterised by higher speed, lower tortuosity, shallow mean and relative depths, high portion of time at the surface (<5 m), and low portion of time in deep water (> 200 m) (Fig 3). The pelagic state was characterised by intermediate values for all data streams while the deep state was characterised by low speed, high tortuosity, greatest mean and relative dive depth, greatest portion of time in deep waters, and least portion of time at the surface (Fig 3). In addition to the data streams, state probabilities were found to be affected by date, bathymetry, seafloor slope, distance to shore, and time of day (Fig 4). The predicted habitat associations and states captured the spatial structure of pelagic and foraging dives (Fig 8).

It is important to note that in the traditional HMM process, the observed data are assumed to stem from a discrete state process, where each state corresponds to a specific behaviour [48]. However, animal behaviour is often hierarchical (i.e., coarse behaviours are composed of finer-scale behaviours), and manifest at different spatio-temporal resolutions, making the biological interpretation of each state a key challenge in HMMs [47–51]. The three behavioural states we interpreted are likely composed of finer-scale behaviours exhibited by narwhal at different temporal and spatial scales. For example, periods of time spent on the surface may include behaviours such as travelling, social activities, or post-dive recovery following deep or energetically costly dives. Further, there may be several transitions among these fine-scale behaviours within the 6-hour steps, and the start and end of the fine-scale behaviours may also occur in different steps (i.e., start at the end of one 6-hour step, and end within the next 6-hour step). Knowing this, the three states represent higher-order “meta-behaviours” depending on the frequency and duration of time spent in finer-scale behavioural states and there is likely some degree of overlap in what the states represent biologically. To help inform the biological interpretation of our states, we also relied on previous knowledge of prey distributions across southern BB (from 2009–2012 to recent) to link behaviours to prey types and are based on individual behaviour of the tagged narwhal, since social groups were not tagged together.

### Narwhal diving behaviours and winter prey preference

Winter is an intense feeding period for narwhal and previous studies suggest that changes in winter diving behaviour, specifically diving depth [5,9,11,22], is linked to seasonal distribution and migration of their primary benthic prey, Greenland halibut [26,27,52–54]. Similar to Watt et al. [9] our results showed that the tagged narwhals decreased their surface behaviours and spent more time diving, while also increasing diving depths as the season progressed (Fig 4a). While narwhals appear to favour deep diving in winter, we also observed individuals adjusting their diving behaviours (i.e., foraging strategies) seasonally and depending on their location. Given the higher energetic cost of deep diving [5,55] we can only assume narwhals are diving to depths associated with lipid-rich benthic or near-benthic prey [6,8,56] and likely target these areas only when large prey are available. During the winter, the highest densities of large mature halibut prey are most often found below 800 m [27,57]. Laidre et al. [6] used stomach content analysis and identified a variety of age classes of prey consumed. Specifically, the size ranges of prey found in narwhal stomachs suggest they are consuming larger halibut (35–55 cm in length and weighing between 200–1400 grams) and higher quantities of juvenile and adult squid.

Narwhals spent 37% of their time in pelagic behaviours (200–500 m) suggesting that when deep diving is not an option or deep-water prey may not be available, they spend time in the mid-water column, or along the continental shelf, likely searching and foraging on other valued pelagic prey (e.g., squid, cod, shrimp [6,28]). Since the energetic requirements for a narwhal to dive <500 m is significantly less, and without other foraging indicators (e.g., dive type/shape, orientation, acoustics; see [14,32,58]) it is more challenging for us to infer the specific emphasis on foraging in these mid-water areas. Since directed foraging to the bottom requires specialized diving strategies (e.g., gliding, perpendicular dives, and efficient oxygen stores), it is suspected that narwhals likely decide to make a deep dive at or close to the surface [5,14]. As such, frequent diving behaviours found in the mid-water environment (outside of the transiting time to get to deeper water) have a high probability of being related to searching and prey consumption.

Mid-water foraging may have been overlooked or underestimated in previous telemetry studies due to the technological limitations of the tags and nature of the binned dive data (see [5]); how forage dives were defined or classified [9]; or that most often, movement and behavioural metrics have often not been incorporated into assessments of foraging. Further to this, depending on the ice conditions throughout the season narwhals are often confined to regions with open leads and cracks, which could restrict certain foraging strategies, such as making long-distance movements to search for other prey patches [11,22]. Different foraging strategies have been observed in other narwhal populations [23], specifically in East Greenland, where narwhals were observed to exhibit ‘transit foraging’, which was characterized by faster and more straight-line horizontal movement during a dive, lower buzzing rates, and deeper target depths, versus ‘stationary foraging’, which was characterized by slower, more tortuous horizontal movements during a dive, high buzzing rates, and shallower target depths. The authors suggested that these different foraging strategies were likely driven by the spatial distribution and density of prey types [14].

The duration and depth of diving behaviour are limited by oxygen stores, which are depleted during dives, and must be replenished during post-dive rest periods at the surface [14]. For narwhals, the energetic cost of a dive increases with both its duration, speed, and intensity of stroke [14,55]. Therefore, we predicted that deeper, more strenuous dives would be associated with longer surface recovery times. In support of this hypothesis, we observed that within the pelagic and deep diving states mean relative depth increased as bathymetry increased (Fig 3d(i)), and the mean surface time also increased (Fig 3f(ii)). However, when comparing between states, mean surface time was longer in the pelagic state than the deep state (Fig 3f), suggesting that the pelagic state may include a larger frequency of surface behaviours, outside of post-dive resting, than in the deep diving state. However, the duration of post-dive surface activity does appear to increase with relative dive depth. A weak relationship between post-dive duration and dive depth has also been shown in other studies [21,59], suggesting that either these tagged individuals did not reach their aerobic limit [55] on deeper dives, resulting in less time needed at the surface, or that the post-dive response may be capturing other behaviours not recognized or known [59] (these could include socialising, mating, shallow less-energetic foraging, and rest behaviours not associated with dive recovery). In our case, surface behaviour was defined between 6 and 200 m, potentially weaning out some post-dive rest; however, depending on the individual and environmental conditions, studies have shown that post-dive behaviours can occur at a variety of shallow depths [59], and narwhals are also known to glide passively between 50 and 250 m to reduce activity between deep forage dives [14,55].

The time of day impacted all three behaviours, with the probability of pelagic diving being the highest at midnight and declining into the early morning, while deep diving behaviours were more prevalent in the late day-evening (Fig 4e). It is important to note that due to programming of dive collections, we only had four discrete points per day to reference these behaviours and no dive profile to evaluate specific state transitions. However, we suspect these observations could be linked to certain prey species undertaking diurnal vertical migrations. This has been observed both in halibut [52,60–62] and squid spp. [63,64], which tend to stay deeper during the day (i.e., to avoid predators, and for spawning and temperature responses). The exact age classes of halibut that exhibit diurnal behaviour is not entirely known. Ngô et al. [59] was able to show diurnal effects on the transition probabilities and interestingly, over a 12 hour period, the transition probabilities of the “forage-type” dives (>350 m) were most dependent on the time of day and peaked at night.

### Narwhal space use and links to foraging habitat

Narwhals are well adapted to their winter ice habitat (characterized by dense pack ice, fast ice, and offshore leads) [17,18], yet ice conditions in BB can have significant inter-annual variation due to factors such as ocean currents, air temperature, and weather patterns [65]. Sea-ice formation starts in BB during October and covers almost all of the area by March, followed by the onset of the melting season in April, as the sea ice retreats westward [65]. Migration movements and phenology can vary across individuals, but once narwhals become ‘resident’ in the overwintering area they tend to stay in large groups, often select areas with available open water [11,18,32], and exhibit lower move-persistence in areas with higher sea ice coverage, potentially related to navigation or foraging [42]. Kenyon et al. [11] examined BB narwhal winter habitat use and environmental conditions with this same dataset and found that habitat selection was not significantly driven by ice conditions (i.e., ice concentration, thickness, and floe size), therefore, sea ice variables were not included in our model. The winter dates for our analysis were manually selected for each individual and in some cases, we did not observe a strong decrease in surface time or clear cessation of southward movement, which signalled arrival to the overwintering area. Therefore, the defined winter periods may still include some migration behaviour and may have resulted in more surface behaviours for some individuals early in the season (see Fig 3f and 5a).

Spatially the overwintering area features diverse seafloor habitats, including deep polar waters (up to ~2500 m), continental shelves (500–700 m) along Baffin Island and West Greenland, and shallow sills at both ends of BB, forming a semi-enclosed basin with Arctic and Atlantic inputs [65]. The Davis Strait sill, though not a continental shelf, has similar depths (600–900 m). We observed an increase in surface behaviours in areas with steep seafloor slopes (Fig 4c), particularly along the continental shelf edge, where depths drop rapidly to ~2000 m in central BB. This finding was similar to Shuert et al. [42], who found that narwhal exhibited more transiting type behaviours in the offshore environment along the steep areas of the continental shelf slope. Due to the low levels of light during the winter season, narwhals may be using the topography of the continental shelf and fast ice edges more predominantly for navigation, rather than foraging habitat. The decrease in diving observed could also suggest that the energetic risk of diving at these steep locations, possibly beyond 1500–1800 m (which we only see occasionally in the winter), is too great for narwhals. The edges of continental shelves, where there can be a mixture of shallow and deep water, often experience high productivity driven by factors such as upwelling, ice edge dynamics (i.e., marginal ice zones), and nutrient influx from water circulation [66–68]. However, in the winter decreased light along with dense sea ice cover limits the amount of light penetration, and this may also reduce overall productivity at these zones [69]. In addition, narwhals may be more interested in targeting prey that occupy the bottom of the shelf and not the edge.

The proportion of time narwhals spent in pelagic and deep-diving states was almost equal, although spatially they appear to be focused in different areas. Pelagic diving behaviours were most common in the northern part of the overwintering area (with a band of pelagic behaviours around 68° N) (Fig 6-7b). This high frequency of pelagic diving behaviours seen further north may also indicate site or aggregation specific selection of the water column, a trend also observed by Laidre et al. [5]. Whales tagged from AI and ES tend to follow a similar fall migration route; however, once they reach southern BB, AI whales will stay further north in the overwintering area [2,5,20]. The highest frequency of data used for our model predictions came from this region, contributed by the 2009 AI tagged whales (Fig 6a), and the whales that traveled south of Davis Strait were all tagged in ES from 2010–2012. Laidre et al. [5] also documented shallower diving patterns for whales that stayed in the northern wintering grounds, despite the bathymetry being deeper, suggesting differences in available prey or depths that are either too costly or impossible physiologically for narwhal to reach.

**Fig 10:**
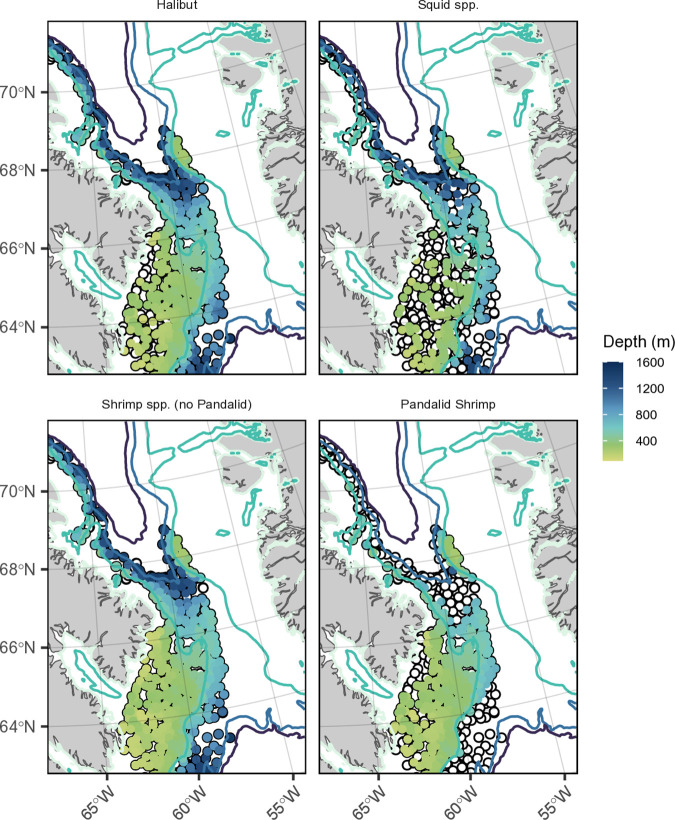
Occurrence of four key winter prey types for Baffin Bay narwhal. The four panels represent Greenland halibut (Halibut), squid species (Squid spp.), various shrimp species (shrimp spp. excluding Pandalid shrimp), and Pandalid shrimp found in multi-species bottom trawl surveys only conducted in Canadian waters (Divisions 0A and 0B) between October and November 2009-2012. Locations of all trawl sets are shown in open black circles and species occurrence coloured in by starting depth (m) of the set. Bathymetric contour lines (light to dark) indicate 0 m, 500 m, 1400 m, and 1800 m. Data (only shown for this study area) was supplied by Fisheries and Oceans Canada Central and Arctic Multi-species Stock Assessment Survey Database [72].

Stable isotope analysis has corroborated the theory of different foraging strategies across narwhal populations and regions [23,24,70,71], and as an indication of possible shrimp-focused foraging [23], we predicted that narwhal diving behaviours in the pelagic state would frequently occur over the continental shelf, with dives often directed to the bottom. Dives directed to the sea floor in the pelagic state were more scattered across the overwintering range than the deep-benthic dives (Fig 9b-c), and dives to 75–100% of the total depth only occurred occasionally. However, a few recurrent areas were observed on the west side of Davis Strait (close to Baffin Island) and along the Greenland shelf, west of Disko Bay. Shrimp tend to occupy sandy bottoms on the continental shelf or slope (between 200–600) and have been found in high numbers in the North Atlantic Fisheries Organization (NAFO) multi-species bottom trawl surveys conducted only in Canadian waters (Divisions 0A and 0B) (Fig 10) [72–76]. Fulton et al. [73] described high densities of *P. borealis* just south of Davis Strait, close to southern Baffin Island, noting habitat suitability is driven primarily by oceanographic conditions, specifically water temperature, present in the area. Interestingly, halibut can also be found in the lower to mid-water column pelagic environment, and both diel and seasonal cycles of vertical migrations (driven primarily by prey availability, temperature, and predator avoidance) have been recorded [60,77].

Deep diving behaviours were more common in the southern portion of the overwintering area (Figs 6b and7c) and narwhals that spent the most time in this area were all tagged in ES in 2010–2012. As expected, the probability of the deep diving state occurring increased in regions with increased bathymetry (Fig 4b) and deep benthic-focused dives, typically within 50–100% of the bottom, were most common in the central areas of southern BB (outside of the 500 m isobath), along the Greenland shelf, and just south of Davis Strait (Fig 9). Similarly, Laidre et al. [22] described narwhal taking focused dives to the bottom, most often in deep water, and correlated this behaviour with variations in biomass and length frequency distributions of Greenland halibut across the wintering area. The NAFO trawl surveys found halibut throughout the southern overwintering area (Fig 10), with large densities observed just north of Davis Strait [27]. The Davis Strait region (inshore and offshore areas) has been identified as an important habitat for all age classes of Greenland halibut, specifically mature adults, with a presumed spawning ground along the shallow water ridge between Baffin Island and Greenland (thought to occur between December and March south of 67° N) [54,78]. Squid, although not a benthic species, can be found from mid-water depths (~400 m) up to depths of 1400 m [22,79] and are common in Canadian waters (Fig 10) and on the West Greenland shelf [80]. Although we did not complete a post-hoc analysis linking the inferred behavioural states to specific prey types shown in Fig 10, due to sparse data and temporal inconsistencies, future studies that can collect this information concurrently could more precisely explore the spatial relationship between narwhal foraging behaviours and prey distribution.

The overall minimum Arctic sea ice extent in 2010–2011 was one of the lowest on record at the time, specifically BB-Davis Strait remained ice free until November, and ice only covered the wintering area by mid-December [11]. Although we only have four years to compare, it is conceivable that seasonal ice conditions will restrict access to certain feeding grounds and that during lower ice years narwhals are able to make broader movements, increasing the likelihood of horizontal foraging trips, and opportunistic selection for mid-water or pelagic-benthic prey patches, like shrimp, not regularly targeted when heavy ice is present. Changes in seasonal ice conditions and declines in sea ice across the region have been linked to narwhal prey switching within [81] and across [9] individuals. The delayed ice formation and lower sea ice extent was likely the catalyst of broader movement patterns and more transiting behaviours observed in 2010–2011. Shuert et al. [42] also found that individuals spent more time transiting in years when ice extent was lower. Most travelling to new areas occurs at the surface [82] and as mentioned, two narwhals (51871 and 51872) showed extensive transiting behaviour in 2010, particularly 51872, a young male (est. 15–16 years based on tusk length-age ratio; see [20,83]) that migrated towards Greenland. One theory could be that male juvenile narwhals may wander more or exhibit a “bold” gene. This concept of ‘boldness’ has been documented and described for other migratory species such as beluga whales (*Delphinapterus leucas*) [84], polar bears (*Ursus maritimus*) [47], and songbirds [85]; however, research on this as it pertains to narwhal is limited.

### Historical data challenges and limitations of behavioural state predictions

A key challenge of the Argos data used in this study was not only that it contained high location error, but that the tags were programmed at a fixed interval, with large gaps due to duty cycling. This combination resulted in data displaying both fixed and irregular characteristics, as duty cycling imposes fixed sampling rates interspersed with lengthy, irregular gaps, and uneven time interval of the dive collections. This mixed nature complicated modeling efforts, especially given that many modern movement models (e.g., HMMs, movement persistence models, step-selection functions) often assume the data is of a single structure. The binned nature of the dive data, and coarser (6-hour) temporal resolution, presented further challenges in model building and interpretation of results, particularly the inconsistencies in tag programming from 2009 to 2010–2012 (see S3 Appendix in S1 File). Steps were taken throughout the data processing and model building process to improve the predicted location estimates and the quality and accuracy of behavioural state predictions, such as using re-analysed locations and updated error model locations (i.e., Lowther et al. [38] showed that 95% of all Argos location errors were within 4.2 km from their true position), aggregating all movement tracks from summer and winter, removing large gaps of no data, and incorporating different dive data streams to differentiate between pelagic and deep water environments targeted by narwhal. Multiple imputation methods that account for observation error attributed to temporally-irregular or missing data [86] were considered but not used due to concerns that high variability in the numerous imputed steps would cause the models to capture imputation patterns rather than true movement. Nonetheless, we acknowledge that there remains some degree of error in the predicted paths (and the corresponding covariate values) used in our analysis; however, our results make biological sense and are consistent with previous studies.

We also recognize that dives may not always be directed towards specific depths, and foraging time may include searching at multiple depths for different prey patches [5]. This is one of the pitfalls of histogram-structured (binned) data, which can mask these behaviours and potentially show that individuals are not selective in their utilization of the water column if they dive to the bottom in habitats with different bathymetric structure [5]. Given this, we incorporated bathymetry as an effect on just the diving behaviours (pelagic and deep states), creating a hard limit to an individual’s diving depth at a given location. Nevertheless, dive behaviours defined as primarily pelagic in nature may still include a portion of time when the whales go beyond 400 m, if the bathymetry allows. High resolution GPS tags that collect non-binned diving data, including maximum depth, or independent dive categories, would better capture the fine-scale behaviours (such as dive type and shape, orientation, acoustics, etc.) required for a complete assessment of foraging behaviours across seasons (see [58,59,87,88]).

## Conclusions

This study utilized an older telemetry dataset to extend our knowledge of narwhal winter behaviour and space use. This dataset is unique in that it is the only time series to date that has lasted well into the winter season for the BB population, and along with only a few movement analyses of this nature for narwhals of different populations (e.g., [59]), none have described winter behaviours across years for so many individuals. Despite the computational challenges and complexities of analysing irregular movement data with HMMs, we were able to identify important locations and patterns (and specific environmental conditions) across the overwintering area where narwhals exhibit both deep and pelagic diving behaviours, and periods of reduced diving. Further, the spatial and temporal differences in these diving strategies across the winter range suggests that narwhals likely target different prey types depending on the bathymetry and topography of the area, and cycle of the winter season. Since it is hypothesized that narwhal consume most of their high-energy prey in the winter season, documenting any changes to winter foraging strategies (specifically at which depths prey removal may occur) is important for enhancing our current understanding of how narwhal may adapt with climate change (e.g., [31]), anthropogenic disturbance (e.g., [89]), and emergence of new species on the wintering ground (e.g., [73]). The methods developed here for handling the duty cycling of error-prone location data could be adapted for other older or historical telemetry datasets, which are often difficult to use with newer modeling frameworks. With technological developments in tag design and remote tagging methods there is hope that researchers will continue collecting more up to date and fine-scale data that can focus on winter foraging and movement of narwhal in a rapidly changing environment.

## Supporting information

S1 File S1 Appendix. Additional details on the telemetry data processing, such a selecting winter dates and filtering out data gaps. S2 Appendix. Additional details on data stream exploration, and selection of final data streams. S3 Appendix. Additional details on early model development, including how the effect of tag programming was included and the overall impact of removing 2009 tags on the state predictions.
**S1 Table. Details of the dive categories collected by the 2009–2012 satellite tags.** Two main dive bin categories were included in this study: Dive Maximum Depth (DMD) and Time at Depth (TAD). There were some inconsistencies in tag programming between the 2009 and 2012 tagging years, resulting in different maximum depths (meters) recorded.
(DOCX)

S1 Fig Defining winter dates for study. Example of definition of winter dates based on standardized value of surface time, and x and y coordinates. Beginning of winter is denoted by the vertical red line and the end of winter is denoted by the vertical blue line.
(TIF)

S2 Fig Observed data gaps of raw narwhal telemetry data. Histogram of observed data gaps of narwhal transmissions, shown as difference in time (hours).
(TIF)

S3 Fig Pre-model testing of different movement data streams. Histograms and autocorrelation functions for seven movement data streams: step length, mean speed, observed mean speed, turning angle, mean angle, observed mean angle, and move persistence (tortuosity).
(TIF)

S4 Fig Pearson’s correlation matrix for movement data streams. Pearson’s correlation matrix for the seven tested movement data streams: step length, mean speed, observed mean speed, turning angle, mean angle, observed mean angle, and move persistence (tortuosity).
(TIF)

S5 Fig Pre-model testing of different dive data streams. Histograms and autocorrelation functions for 11 different dive data streams: Surface, shallow, moderate, deep, mean depth, relative dive depth, max depth, max depth (5%), max depth (10%), max depth (20%), and weighted max depth.
(TIF)

S6 Fig Pearson’s correlation matrix for dive data streams. Correlation matrix for the 11 tested dive data streams: Surface, shallow, moderate, deep, mean depth, relative dive depth, max depth, max depth (5%), max depth (10%), max depth (20%), and weighted max depth.
(TIF)

S7 FigEffect of tag programming on emission probabilities of dive parameters.Predicted effect of tag programming on emission probabilities for the shape (κ), scale (λ), mean (μ), and standard deviation (σ) parameters of three diving data streams: mean depth (Md) and relative dive depth (Rd) and time at depth (Dp). Effects were assumed to be independent for each of the three behaviour states: state 1 (surface activity; green), state 2 (pelagic diving; blue), and state 3 (benthic diving; red). Error bars represent the estimated 95% confidence intervals of the means.(TIF)

S8 Fig Winter distribution of decoded predicted states with just 2010–2012 tag programming. (a) The total number of steps that occurred in each cell (50 km by 50 km), which represents the total data that went into predictions for each cell (higher number of steps used indicates greater confidence in state predictions), and (b) calculated as the state S^‘ with the highest within-cell proportion relative to the frequency of each state across all cells. Cells with < 4 steps are not plotted, and hash lines represent cells with < 16 total steps, which indicate low certainty in those state predictions.
(TIF)

S9 Fig Predicted winter distribution of decoded behavioural states with just 2010–2012 tag programming. Behavioural decoded states (a) 1 (green), (b) 2 (blue), and (c) 3 (red), with dark areas representing high state frequency within each cell (50 km by 50 km). Cells with < 4 steps are not plotted, and hash lines represent cells with <16 total steps.
(TIF)

## References

[1] Heide‐Jørgensen MP, Richard PR, Dietz R, Laidre KL. A metapopulation model for Canadian and West Greenland narwhals. Animal Conservation. 2012;16(3):331–43. doi: 10.1111/acv.12000

[2] Heide-Jørgensen MP, Nielsen NH, Hansen RG, Schmidt HC, Blackwell SB, Jørgensen OA. The predictable narwhal: Satellite tracking shows behavioural similarities between isolated subpopulations. J Zool. 2015;297(1):54–65. doi: 10.1111/jzo.12257

[3] Heide-Jørgensen MP, Laidre KL, Richard PR. Autumn movements, home ranges, and winter density of narwhals (*Monodon monoceros*) tagged in Tremblay Sound, Baffin Island. Polar Biol. 2002;25:331–41. doi: 10.14430/arctic785

[4] Heide-Jørgensen MP, Dietz R, Laidre KL, Richard P, Orr J, Schmidt HC. The migratory behaviour of narwhals (*Monodon monoceros*). Canadian Journal of Zoology. 2003;81(8):1298–305. doi: 10.1139/z03-117

[5] Laidre KL, Heide-Jørgensen MP, Dietz R, Hobbs RC, Jørgensen OA. Deep-diving by narwhals *Monodon monoceros*: Differences in foraging behavior between wintering areas? Mar Ecol Prog Ser. 2003;261:269–81. doi: 10.3354/meps261269

[6] Laidre KL, Heide-Jørgensen MP. Winter feeding intensity of narwhals (*Monodon monoceros*). Mar Mammal Sci. 2005;21(1):45–57. doi: 10.1098/rsbl.2022.0423

[7] Shuert CR, Marcoux M, Hussey NE, Watt CA, Auger-Méthé M. Assessing the post-release effects of capture, handling and placement of satellite telemetry devices on narwhal (*Monodon monoceros*) movement behaviour. Conserv Physiol. 2020;9(1):1–16. doi: 10.1093/conphys/coaa128 PMC7905160 33659061

[8] Watt CA, Orr JR, Ferguson SH. Spatial distribution of narwhal (*Monodon monoceros*) diving for Canadian populations helps identify important seasonal foraging areas. Can J Fish Aquat Sci. 2017;95:41–50. doi: 10.1139/cjz-2016-0178

[9] Watt CA, Orr JR, Heide-Jørgensen MP, Nielsen NH, Ferguson SH. Differences in dive behaviour among the world’s three narwhal *Monodon monoceros* populations correspond with dietary differences. Mar Ecol Prog Ser. 2015;525:273–85. doi: 10.3354/meps11202

[10] Dietz R, Heide-Jørgensen MP, Richard P, Orr J, Laidre K, Schmidt HC. Movements of narwhals (*Monodon monoceros*) from Admiralty Inlet monitored by satellite telemetry. Polar Biol. 2008;31(1):1295–306. doi: 10.1139/z95-248

[11] Kenyon KA, Yurkowski DJ, Orr J, Barber D, Ferguson SH. Baffin Bay narwhal (*Monodon monoceros*) select bathymetry over sea ice during winter. Polar Biology. 2018;41(10):2053–63. doi: 10.1007/s00300-018-2345-y

[12] Shuert CR, Marcoux M, Hussey NE, Heide-Jørgensen MP, Dietz R, Auger-Méthé M. Decadal migration phenology of a long-lived Arctic icon keeps pace with climate change. Proceedings of the National Academy of Sciences of the United States of America. 2022;119(45):1–8. doi: 10.1073/pnas.2121092119 PMC9659343 36279424

[13] Finley K. The estuarine habitat of the beluga or white whale, Delphinapterus leucas. Cetus. 1982;4(2):4–5.

[14] Tervo OM, Ditlevsen S, Ngô MC, Nielsen NH, Blackwell SB, Williams TM. Hunting by the stroke: How foraging drives diving behavior and locomotion of East-Greenland narwhals (*Monodon monoceros*). Frontiers in Marine Science. 2021;7:1–18. doi: 10.3389/fmars.2020.00001

[15] Dietz R, Heide-Jørgensen MP, Richard PR, Acquarone M. Summer and fall movements of narwhals (*Monodon monoceros*) from northeastern Baffin Island towards northern Davis Strait. Arctic. 2001;54(3):244–61. doi: 10.14430/arctic785

[16] DFO. Harvest allocation modelling for Baffin Bay narwhal (*Monodon monoceros*) stocks. 2021/001. 2021.

[17] Koski W, Davis R. Distribution and numbers of narwhals (*Monodon monoceros*) in Baffin Bay and Davis Strait. Bioscience. 1994;39:15–40. doi: 10.1139/f91-038

[18] Laidre KL, Heide-Jørgensen MP. Life in the lead: Extreme densities of narwhals *Monodon monoceros* in the offshore pack ice. Mar Ecol Prog Ser. 2011;423:269–78. doi: 10.3354/meps08941

[19] Stephenson SA, Hartwig L. The Arctic Marine Workshop: Freshwater Institute Winnipeg, Manitoba, February 16-17, 2010. 2010.

[20] Watt CA, Orr JR, Leblanc B, Richard PR, Ferguson SH. Satellite tracking of narwhals (*Monodon monoceros*) from Admiralty Inlet (2009) and Eclipse Sound (2010-2011). DFO Can Sci Advis Secr Res Doc. 2012;2012(46):iii–17.

[21] Laidre KL, Heide-Jørgensen MP, Dietz R. Diving behavior of narwhals (*Monodon monoceros*) at two coastal localities in the Canadian High Arctic. Can J Zool. 2002;80:624–35. doi: 10.1139/z02-041

[22] Laidre KL, Heide-Jørgensen MP, Jørgensen OA, Treble MA. Deep-ocean predation by a high Arctic cetacean. ICES J Mar Sci. 2004;61(3). doi: 10.1016/S1054-3139(04)00011-6

[23] Watt CA, Heide-Jørgensen MP, Ferguson SH. How adaptable are narwhal? A comparison of foraging patterns among the world’s three narwhal populations. Ecosphere. 2013;4(6):1–15.

[24] Watt CA, Ferguson SH. Fatty acids and stable isotopes (δ13C and δ15N) reveal temporal changes in narwhal (*Monodon monoceros*) diet linked to migration patterns. Mar Mammal Sci. 2015;31(1):21–44. doi: 10.1111/mms.12131

[25] Treble MA. Report on Greenland halibut caught during the 2015 trawl survey in Divisions 0A and 0B. 25. 2016.

[26] Jørgensen OA. Bottom trawl survey in Baffin Bay, NAFO Divisions 1A, 2010. NAFO SCR. 2011;11(010):1–14.

[27] Hedges KJ. Report on Greenland halibut (*Reinhardtius hippoglossoides*) caught during the 2022 trawl survey in Subarea 0. NAFO SCR Doc. 2023;23(029):1–16.

[28] Watt CA, Ferguson SH, Fisk A. Using stable isotope analysis as a tool for narwhal (*Monodon monoceros*) stock delineation. DFO Can Sci Advis Sec Res Doc. 2012;2012(057):iv+29. doi: 10.1111/j.1748-7692.2009.00354.x

[29] Stroeve JC, Markus T, Boisvert L, Miller J, Barrett AP. Changes in Arctic melt season and implications for sea ice loss. Geophys Res Lett. 2014;41:1216–25. doi: 10.1002/2013GL058951

[30] Laidre KL, Stirling I, Lowry LF, Wiig O, Heide-Jørgensen MP, Ferguson SH. Quantifying the sensitivity of Arctic marine mammals to climate-induced habitat change. Ecol Appl. 2008;18(2 Suppl):S97-125. doi: 10.1890/06-0546.1 18494365

[31] Chambault P, Tervo OM, Garde E, Hansen RG, Blackwell SB, Williams TM, et al. The impact of rising sea temperatures on an Arctic top predator, the narwhal. Sci Rep. 2020;10(1):18678. doi: 10.1038/s41598-020-75658-6 33122802 PMC7596713

[32] Heide-Jørgensen MP, Blackwell SB, Williams TM, Sinding MHS, Skovrind M, Tervo OM, et al. Some like it cold: Temperature-dependent habitat selection by narwhals. Ecol Evol. 2020;10(15):8073–90. doi: 10.1002/ece3.6464 32788962 PMC7417212

[33] Chimienti M, Blasi MF, Hochscheid S. Movement patterns of large juvenile loggerhead turtles in the Mediterranean Sea: Ontogenetic space use in a small ocean basin. Ecol Evol. 2020;10(14):6978–92. doi: 10.1002/ece3.6370 32760506 PMC7391346

[34] Jakobsson M, Mayer LA, Bringensparr C, Castro CF, Mohammad R, Johnson P, et al. The International Bathymetric Chart of the Arctic Ocean Version 4.0. Sci Data. 2020;7(1):176. doi: 10.1038/s41597-020-0520-9 32647176 PMC7347603

[35] Orr JR, Joe R, Evic D. Capturing and handling of white whales (*Delphinapterus leucas*) in the Canadian arctic for instrumentation and release. Arctic. 2001;54(3):299–304. doi: 10.14430/arctic789

[36] Heide-Jørgensen MP, Dietz R, Laidre KL, Nicklen P, Garde E, Richard P. Resighting of a narwhal (*Monodon monoceros*) instrumented with a satellite transmitter. Arctic. 2008;61(4):395–8.

[37] Boyd JD, Brightsmith DJ. Error properties of Argos satellite telemetry locations using least squares and Kalman filtering. PLoS One. 2013;8(5):e63051. doi: 10.1371/journal.pone.0063051 23690980 PMC3656847

[38] Lowther AD, Lydersen C, Fedak MA, Lovell P, Kovacs KM. The Argos-CLS Kalman Filter: Error Structures and State-Space Modelling Relative to Fastloc GPS Data. PLoS One. 2015;10(4):e0124754. doi: 10.1371/journal.pone.0124754 25905640 PMC4408085

[39] McClintock BT, London JM, Cameron MF, Boveng PL. Modelling animal movement using the Argos satellite telemetry location error ellipse. Methods Ecol Evol. 2015;6:266–77.

[40] R Core Team. R: A language and environment for statistical computing. 2023. Available from: https://www.r-project.org//

[41] Freitas C. Argosfilter: Argos Locations Filter [Internet]. 2022. Available from: https://cran.r-project.org/package=argosfilter

[42] Shuert CR, Hussey NE, Marcoux M, Heide-Jørgensen MP, Dietz R, Auger-Méthé M. Divergent migration routes reveal contrasting energy-minimization strategies to deal with differing resource predictability. Mov Ecol. 2023;11(1):31. doi: 10.1186/s40462-023-00397-y 37280701 PMC10245675

[43] McClintock BT, Michelot T. momentuHMM: R package for generalized hidden Markov models of animal movement. Methods Ecol Evol. 2018;9(6):1518–30.

[44] Johnson DS, London JM, Lea M-A, Durban JW. Continuous-time correlated random walk model for animal telemetry data. Ecology. 2008;89(5):1208–15. doi: 10.1890/07-1032.1 18543615

[45] GEBCO Compilation Group. GEBCO 2023 Grid: A continuous terrain model of the global oceans and land. 2023. doi: 10.5285/f98b053b-0cbc-6c23-e053-6c86abc0af7b

[46] Zucchini W, MacDonald IL, Langrock R. Hidden Markov Models for Time Series: An Introduction Using R. Second ed. Boca Raton, FL: Chapman & Hall/CRC. 2016.

[47] Togunov RR, Derocher AE, Lunn NJ, Auger-Méthé M. Drivers of polar bear behavior and the possible effects of prey availability on foraging strategy. Mov Ecol. 2022;10(1):50. doi: 10.1186/s40462-022-00351-4 36384775 PMC9670556

[48] McClintock BT, Johnson DS, Hooten MB, Hoef JMV, Morales JM. When to be discrete: The importance of time formulation in understanding animal movement. Mov Ecol. 2014;2(21):1–14. doi: 10.1186/s40462-014-0021-6 25709830 PMC4337762

[49] Leos-Barajas V, Michelot T. An introduction to animal movement modeling with hidden Markov models using Stan for Bayesian inference. arXiv. 2018. 10639.

[50] McClintock BT, Langrock R, Gimenez O, Cam E, Borchers DL, Glennie R, et al. Uncovering ecological state dynamics with hidden Markov models. Ecol Lett. 2020;23(12):1878–903. doi: 10.1111/ele.13610 33073921 PMC7702077

[51] Adam T, Griffiths CA, Meese EN, Lowe CG, Blackwell PG, Righton D. Joint modelling of multi-scale animal movement data using hierarchical hidden Markov models. Methods Ecol Evol. 2019;2019(10):1536–50. doi: 10.1111/2041-210X.13241

[52] Jørgensen O. Movement patterns of Greenland halibut, Reinhardtius hippoglossoides (Walbaum), at West Greenland, as inferred from trawl survey distribution and size data. J Northwest Atl Fish Sci. 1997;21:23–37. doi: 10.2960/J.v43m677

[53] Treble MA. Report on Greenland halibut caught during the 2014 trawl survey in Divisions 0A and 0B. NAFO SCR Doc. 2015;16(025):1–14.

[54] Vihtakari M, Elvarsson BP, Treble M, Nogueira A, Hedges K, Hussey NE. Migration patterns of Greenland halibut in the North Atlantic revealed by a compiled mark-recapture dataset. ICES J Mar Sci. 2022;79(6):1902–17. doi: 10.1093/icesjms/fsac127

[55] Williams TM, Davis RW, Fuiman LA, Francis J, Le Boeuf BJ, Horning M, et al. Sink or swim: Strategies for cost-efficient diving by marine mammals. Science. 2000;288(5463):133–6. doi: 10.1126/science.288.5463.133 10753116

[56] Lawson JW, Magalhães AM, Miller EH. Important prey species of marine vertebrate predators in the northwest Atlantic: Proximate composition and energy density. Mar Ecol Prog Ser. 1998;164:13–20. doi: 10.3354/meps291043

[57] Bowering WR, Nedreaas KH. A comparison of Greenland halibut (*Reinhardtius hippoglossoides (Walbaum)*) fisheries and distribution in the Northwest and Northeast Atlantic. Sarsia. 2000;85(1):61–76. doi: 10.1080/00364827.2000.10414555

[58] Blackwell SB, Tervo OM, Conrad AS, Sinding MHS, Hansen RG, Ditlevsen S, et al. Spatial and temporal patterns of sound production in East Greenland narwhals. PLoS One. 2018;13(6):e0198295. doi: 10.1371/journal.pone.0198295 29897955 PMC5999075

[59] Ngô MC, Heide-Jørgensen MP, Ditlevsen S. Understanding narwhal diving behaviour using Hidden Markov Models with dependent state distributions and long range dependence. PLoS Comput Biol. 2019;15(3):e1006425. doi: 10.1371/journal.pcbi.1006425 30870414 PMC6417660

[60] Boje J, Neuenfeldt S, Sparrevohn CR, Eigaard O, Behrens JW. Seasonal migration, vertical activity, and winter temperature experience of Greenland halibut *Reinhardtius hippoglossoides* in West Greenland waters. Mar Ecol Prog Ser. 2014;508:211–22. doi: 10.3354/meps10874

[61] Boje J, Hareide N. Trial deepwater longline fishery in the Davis Strait, May-June 1992. NAFO SCR Doc. 1993;93/53(N2236):6.

[62] Vollen T, Albert O. Pelagic behavior of adult Greenland halibut (*Reinhardtius hippoglossoides*). Fish Bull. 2008;106:457–70.

[63] Kristensen T. Biology of Squid Gonatus fabricii (*Lichtenstein, 1818*) from West Greenland waters. Vol. 13, Meddelelser om Grønland: Bioscience. Museum Tusculanum Press; 1984. 20 p.

[64] Golikov AV, Ceia FR, Hoving HJT, Queirós JP, Sabirov RM, Blicher ME, et al. Life History of the Arctic Squid Gonatus fabricii (*Cephalopoda: Oegopsida*) Reconstructed by Analysis of Individual Ontogenetic Stable Isotopic Trajectories. Animals (Basel). 2022;12(24):3548. doi: 10.3390/ani12243548 36552473 PMC9774963

[65] Tang CCL, Ross CK, Yao T, Petrie B, Detracey BM, Dunlap E. The circulation, water masses and sea-ice of Baffin Bay. Prog Oceanogr. 2004;63:183–228. doi: 10.1016/j.pocean.2004.09.005

[66] Burgers TM, Tremblay J-É, Else BGT, Papakyriakou TN. Estimates of net community production from multiple approaches surrounding the spring ice-edge bloom in Baffin Bay. Elem Sci Anth. 2020;8(1). doi: 10.1525/elementa.013

[67] Ribeiro CG, Lopes dos Santos A, Trefault N, Marie D, Lovejoy C, Vaulot D. Arctic phytoplankton microdiversity across the marginal ice zone: Subspecies vulnerability to sea-ice loss. Elem Sci Anth. 2024;12(1). doi: 10.1525/elementa.2023.00109

[68] Morán XAG, Lopez-Urrutia A, Calvo-Diaz A, Li WKW. Increasing importance of small phytoplankton in a warmer ocean. Global Change Biology. 2010;16:1137–44. doi: 10.1111/j.1365-2486.2009.01960.x

[69] Leu E, Mundy CJ, Assmy P, Campbell K, Gabrielsen TM, Gosselin M. Arctic spring awakening – steering principles behind the phenology of vernal ice algal blooms. Prog Oceanogr. 2015;139:151–70.

[70] Hobson KA, Fisk A, Karnovsky N, Holst M, Gagnon J m a r c, Fortier M. A stable isotope (d13C, d15N) model for the North Water food web: Implications for evaluating trophodynamics and the flow of energy and contaminants. Deep Sea Res Part II Top Stud Oceanogr. 2002;49:5131–50. doi: 10.1016/S0967-0645(02)00182-0

[71] Dietz R, Riget F, Hobson KA, Heide-Jørgensen MP, Møller P, Cleemann M, et al. Regional and inter annual patterns of heavy metals, organochlorines and stable isotopes in narwhals (*Monodon monoceros*) from West Greenland. Sci Total Environ. 2004;331(1–3):83–105. doi: 10.1016/j.scitotenv.2004.03.041 15325143

[72] DFO. Published by OBIS. Central and Arctic Multi-Species Stock Assessment Surveys Version 6 In OBIS Canada Digital Collections. Bedford Institute of Oceanography, Dartmouth, NS, Canada. Published by OBIS. 2016. Available from: http://www.iobis.org/

[73] Fulton S, Walkusz W, Atchison S, Cyr F. Information to support the assessment of Northern Shrimp, *Pandalus borealis*, and Striped Shrimp, *Pandalus montagui*, in the Eastern and Western Assessment Zones, February 2023. DFO Can Sci Advis Sec Res Doc. 2024;2024(016):iv+51.

[74] Baker KD, Mullowney DRJ, Fulton S. Spatiotemporal modelling of northern shrimp Pandalus borealis distribution patterns throughout Canada’s subarctic and arctic regions. Mar Ecol Prog Ser. 2024;740:79–93. doi: 10.3354/meps14651

[75] DFO. An assessment of northern shrimp (*Pandalus borealis*) in shrimp fishing areas 4–6 and of striped shrimp (*Pandalus montagui*) in shrimp fishing area 4 in 2018. Vol. 2019/027, Canadian Science Advisory Secretariat Science Advisory Report. 2019.

[76] Wieland K, Siegstad H. Environmental factors affecting recruitment of northern shrimp *Pandalus borealis* in West Greenland waters. Mar Ecol Prog Ser. 2012;469:297–306. doi: 10.3354/meps09794

[77] Giraldo C, Stasko A, Walkusz W, Majewski A, Rosenberg B, Power M. Feeding of Greenland halibut (*Reinhardtius hippoglossoides*) in the Canadian Beaufort Sea. J Mar Syst. 2018;183:32–41. doi: 10.1016/j.jmarsys.2018.03.009

[78] Gundersen AC, Stenberg C, Fossen I, Lyberth B, Boje J, Jørgensen OA. Sexual maturity cycle and spawning of Greenland halibut *Reinhardtius hippoglossoides* in the Davis Strait. J Fish Biol. 2010;77(1):211–26. doi: 10.1111/j.1095-8649.2010.02671.x 20646148

[79] Gardiner K, Dick TA. Arctic cephalopod distributions and their associated predators. Polar Research. 2010;29:209–27. doi: 10.1007/s00227-018-3352-9

[80] Zumholz K, Frandsen RP. New information on the life history of cephalopods off west Greenland. Polar Biol. 2006;29(3):169–78. doi: 10.1007/s00300-005-0036-y

[81] Dietz R, Desforges J-P, Rigét FF, Aubail A, Garde E, Ambus P, et al. Analysis of narwhal tusks reveals lifelong feeding ecology and mercury exposure. Curr Biol. 2021;31(9):2012-2019.e2. doi: 10.1016/j.cub.2021.02.018 33705717

[82] Heide-Jørgensen MP, Hammeken N, Dietz R, Orr J, Richard PR. Surfacing times and dive rates for narwhals (*Monodon monoceros*) and belugas (Delphinapterus leucas). Arctic. 2001;54(2):284–98.

[83] Garde E, Heide-Jørgensen MP, Ditlevsen S, Hansen SH. Aspartic acid racemization rate in narwhal (*Monodon monoceros*) eye lens nuclei estimated by counting of growth layers in tusks. Polar Res. 2012;31(1):15865. doi: 10.3402/polar.v31i0.15865

[84] Hill HM, Woodruff MJ, Noonan M. Individual differences in the behavioral characteristics of beluga whales (*Dephinapterus leucas*). Behav Processes. 2019;166:103885. doi: 10.1016/j.beproc.2019.06.008 31185265

[85] Ralph J. Age ratios and their possible use in determining autumn routes of passerine migrants. Wildlife Society Bulletin. 1981;93(2):164–88.

[86] McClintock BT. Incorporating telemetry error into hidden Markov models of animal movement using multiple imputation. J Agric Biol Environ Stat. 2017;22(3):249–69. doi: 10.1007/s13253-017-0285-6

[87] Storrie L, Loseto LL, Sutherland EL, MacPhee SA, O’Corry-Crowe G, Hussey NE. Do beluga whales truly migrate? Testing a key trait of the classical migration syndrome. Mov Ecol. 2023;11(1):53. doi: 10.1186/s40462-023-00416-y 37649126 PMC10469428

[88] Storrie L, Hussey NE, MacPhee SA, O’Corry-Crowe G, Iacozza J, Barber DG. Year-round dive characteristics of male beluga whales from the Eastern Beaufort Sea population indicate seasonal shifts in foraging strategies. Frontiers in Marine Science. 2022;8(715412):1–22. doi: 10.3389/fmars.2021.71541235273967

[89] Tervo OM, Blackwell SB, Ditlevsen S, Garde E, Hansen RG, Samson AL, et al. Stuck in a corner: Anthropogenic noise threatens narwhals in their once pristine Arctic habitat. Sci Adv. 2023;9(30):eade0440. doi: 10.1126/sciadv.ade0440 37494430 PMC10371008

