# Supplementary material for: Behavioural analysis of multi-year satellite telemetry data provides insight into narwhal (Monodon monoceros) winter prey selection in Baffin Bay
Source: PLoS One. 2025 Sep 2;20(9):e0330928. doi: 10.1371/journal.pone.0330928 (PMC12404367; doi:10.1371/journal.pone.0330928)
Supplement: S1 File — S1 Appendix. Additional details on the telemetry data processing, such a selecting winter dates and filtering out data gaps. S2 Appendix. Additional details on data stream exploration, and selection of final data streams. S3 Appendix. Additional details on early model development, including how the effect of tag programming was included and the overall impact of removing 2009 tags on the state predictions. S1 Table. Details of the dive categories collected by the 2009–2012 satellite tags. Two main dive bin categories were included in this study: Dive Maximum Depth (DMD) and Time at Depth (TAD). There were some inconsistencies in tag programming between the 2009 and 2012 tagging years, resulting in different maximum depths (meters) recorded. (DOCX) [file pone.0330928.s001.docx]

# **Supporting Information**

# **S1 Appendix: Telemetry data processing**

**S1 Table.** **Details of the dive categories collected by the 2009-2012 satellite tags**. Two main dive bin categories were included in this study: Dive Maximum Depth (DMD) and Time at Depth (TAD). There were some inconsistencies in tag programming between the 2009 and 2012 tagging years, resulting in different maximum depths (meters) recorded.

| **Tagging year** | **Depth bin category** | **Depth bin (meters)** |
| --- | --- | --- |
| 2009 | DMD | 6, 8, 10, 12, 15, 20, 25, 50, 100, 200, 300, 400, 500, and > 500 |
| 2009 | TAD | 1, 2, 3, 4, 6, 8, 10, 12, 20, 36, 50, 100, 200, >200 |
| 2010 and 2011 | DMD | 6, 8, 10, 12, 15, 20, 100, 200, 400, 800, 1000, 1400, 1800 and >1800 |
| 2010 and 2011 | TAD | 1, 2, 3, 4, 5, 20, 100, 200, 400, 800, 1000, 1400, 1800, and >1800 |
| 2012 | DMD | 6, 8, 10, 12, 15, 20, 100, 200, 400, 800, 1000, 1400 |
| 2012 | TAD | 1, 2, 3, 4, 5, 20, 100, 200, 400, 800, 1000, 1400 |

## **Selecting winter dates**

**S1 Fig.** **Defining winter dates for study.** Example of definition of winter dates based on standardized value of surface time, and x and y coordinates. Beginning of winter is denoted by the vertical red line and the end of winter is denoted by the vertical blue line.

## **Removing large gaps**

Including the programed duty cycling, there were many periods where the tags transmitted intermittently. For example, narwhal tag 39309 (2009) stopped transmitting for 64 days between January 22 and March 27. This creates analytical challenges as discrete-time locations with no missing covariate data (e.g., bathymetry) are required for the maximum-likelihood hidden Markov model (HMM) analysis. Further, as gaps in location data increase, the most likely locations predicted by the continuous-time correlated random walk (CTCRW) tend to be straightened out and equidistant and may be misclassified as a distinct “behavioural” state by the HMM. Therefore, while all predicted locations were used to approximate the most likely environmental covariates, the movement metrics of any steps during which no Argos locations were obtained were nullified (i.e., set to ‘NA’ prior to fitting HMMs).

Based on the peak in observed data gaps at approximately 2 hours (S2 Fig), the CTCRW model predicted missing locations at 2-hour time steps assuming a bivariate normal model based on the Argos error ellipse [1]. The Argos location error ellipses were oriented toward the x-axis, with mean semi-major axis M = 30596 m (median = 8753 m, SD = 83300), semi-minor axis m = 704 m (median = 296 m, SD = 1265), and orientation c = 91° (median = 91°, SD = 17°). The output from this step was then used to predict the most likely locations for each individual narwhal, from which we calculated step length (i.e., straight-line distance between two successive locations).

**S2 Fig. Observed data gaps of raw narwhal telemetry data.** Histogram of observed data gaps of narwhal transmissions, shown as difference in time (hours).

# **S2 Appendix: Data Stream exploration**

Many data streams were explored in the pre-model development phase. Seven movement data streams were examined: step length, mean speed, observed mean speed, turning angle, mean angle, observed mean angle, and move persistence (tortuosity) (S3-S4 Figs). To match the 6-hour interval of the dive data streams, mean speed was calculated by averaging step length of three consecutive 2-hour steps. Observed mean speed and angle refer to values calculated using observed locations (not predicted).

**S3 Fig.** **Pre-model testing of different movement data streams.** Histograms and autocorrelation functions for seven movement data streams: step length, mean speed, observed mean speed, turning angle, mean angle, observed mean angle, and move persistence (tortuosity).

**S4 Fig**. **Pearson’s correlation matrix for movement data streams.** Pearson’s correlation matrix for the seven tested movement data streams: step length, mean speed, observed mean speed, turning angle, mean angle, observed mean angle, and move persistence (tortuosity).

Eleven dive data streams were initially examined. These included the proportion of time spent at the surface (<6m, defined as not diving), shallow (6-20 m), moderate (20-400 m), or deep (>400 m) water, and the maximum depth where individuals spent at least 5%, 10%, and 20% of their time at that depth or deeper. We also assessed instantaneous maximum depth (m) and pseudo-mean depth (hereafter, ‘mean depth’), which was calculated by averaging the middle TAD bin, weighted by the percent of time spent in that bin. Dive depth relative to the sea floor (hereafter, ‘relative dive depth’), which was the mean depth divided by the maximum bathymetry within 20 km of the estimated location, was also tested in the first phase of model development. Dive data stream histograms and autocorrelation functions, and Pearson’s correlation matrix shown in S5 Fig and S6 Fig, respectively.

**S5 Fig**. **Pre-model testing of different dive data streams.** Histograms and autocorrelation functions for 11 different dive data streams: Surface, shallow, moderate, deep, mean depth, relative dive depth, max depth, max depth (5%), max depth (10%), max depth (20%), and weighted max depth.

**S6 Fig.** **Pearson’s correlation matrix for dive data streams.** Correlation matrix for the 11 tested dive data streams: Surface, shallow, moderate, deep, mean depth, relative dive depth, max depth, max depth (5%), max depth (10%), max depth (20%), and weighted max depth.

# **S3 Appendix: Model Development**

## **Step-wise approach of model selection**

The final HMM was built sequentially, adding data streams, covariates, and covariate effects. Data streams were integrated considering several factors: low correlation with existing data streams, maintenance of high predicted state heterogeneity (i.e., avoiding state probability collapse or altered state definitions), minimal residuals, and distinct state distributions. In the case that more than one distribution would be suitable for a specific data stream, the most appropriate distribution was selected using (AIC) Akaike Information Criterion. The addition of each covariate was individually tested against the base model, and the one-covariate model with the lowest AIC was adopted as the new reference. Subsequent covariates were added iteratively based on the lowest AIC until all were included. This process was repeated until all covariates were included in the model. This approach was used as descending on the number of states is a known challenge with HMM analysis, and conventional approaches using AIC or BIC (Bayesian Information Criterion) for model selection have been known to bias support for a larger number of states [2]. New methods are being developed for selecting the number of states in HMMs (e.g.,[3]), however, these have only been demonstrated for simpler HMMs with fewer data streams, and are not yet implemented in any R packages. Covariate effects were interpreted based on their effect size and by examining the 95% confidence intervals for the approximate stationary state distribution [4], which represents the long-term probabilities of each state if the process were to run indefinitely at constant covariate values (see also [5]).

## **Covariate effects**

**S7 Fig. Effect of tag programming on emission probabilities of dive parameters.** Predicted effect of tag programming on emission probabilities for the shape ($\kappa$), scale ($\lambda$), mean ($\mu$), and standard deviation ($\sigma$) parameters of three diving data streams: mean depth (Md) and relative dive depth (Rd) and time at depth (Dp). Effects were assumed to be independent for each of the three behaviour states: state 1 (surface activity; green), state 2 (pelagic diving/foraging; blue), and state 3 (benthic diving/foraging; red). Error bars represent the estimated 95% confidence intervals of the means.

Formulas for shape and scale for Relative depth:

$\kappa_{S,t}^{\left( Rd \right)}={\{}_{\beta_{0,S}^{\left( Rd \right)}+\beta_{1,S}^{\left( Rd \right)} prog+\beta_{2,S}^{\left( Rd \right)} bath}^{\beta_{0,S}^{\left( Rd \right)}+\beta_{1,S}^{\left( Rd \right)} prog} {}_{Otherwise}^{if S=1}$

$$\lambda_{S,t}^{\left( Rd \right)}={\{}_{\beta_{3,S}^{\left( Rd \right)}+\beta_{4,S}^{\left( Rd \right)} prog+\beta_{5,S}^{\left( Rd \right)} bath}^{\beta_{3,S}^{\left( Rd \right)}+\beta_{4,S}^{\left( Rd \right)} prog} {}_{Otherwise}^{if S=1}$$

Formulas for mean and standard deviation for surface time:

$\mu_{S,t}^{\left( St \right)}={\{}_{\beta_{0,S}^{\left( St \right)}+\beta_{1,S}^{\left( St \right)} wDay+\beta_{2,S}^{\left( St \right)} bath}^{\beta_{0,S}^{\left( St \right)}+\beta_{1,S}^{\left( St \right)} wDay} {}_{Otherwise}^{if S=1}$

$$\sigma_{S,t}^{\left( St \right)}={\{}_{\beta_{3,S}^{\left( St \right)}+\beta_{4,S}^{\left( St \right)} wDay+\beta_{5,S}^{\left( St \right)} bath}^{\beta_{3,S}^{\left( St \right)}+\beta_{4,S}^{\left( St \right)} wDay} {}_{Otherwise}^{if S=1}$$

Formulas for mean and standard deviation for time at depth:

$\mu_{S,t}^{\left( Dp \right)}={\{}_{\beta_{0,S}^{\left( Dp \right)}+\beta_{1,S}^{\left( Dp \right)} prog+\beta_{2,S}^{\left( Dp \right)} bath}^{\beta_{0,S}^{\left( Dp \right)}+\beta_{1,S}^{\left( Dp \right)} prog} {}_{Otherwise}^{if S=1}$

$$\sigma_{S,t}^{\left( Dp \right)}={\{}_{\beta_{3,S}^{\left( Dp \right)}+\beta_{4,S}^{\left( Dp \right)} prog+\beta_{5,S}^{\left( Dp \right)} bath}^{\beta_{3,S}^{\left( Dp \right)}+\beta_{4,S}^{\left( Dp \right)} prog} {}_{Otherwise}^{if S=1}$$

## **State predictions (tag programming just 2010+)**

Below we provide a comparison of the Viterbi state prediction accuracy (only on base model without forward model covariate selection) to demonstrate that state predictions for all years combined (presented in manuscript) do not differ significantly when just 2010-2012 (2010+) data is used, despite the differences in tag programming. State prediction accuracy is around 90% the same between the two models.

# load models
# get a list of all the RDS files in the directory
rds_files <- list.files(model_dir, pattern = "\\.rds$", full.names = TRUE) %>%
 str_subset("allID") %>%
 str_subset("26|18")
# load each file into the workspace and assign it the same name as the file
for(i in rds_files){
 assign(gsub(".rds", "", basename(i)), readRDS(i))
}

# select models to compare
M1 <- HMM_ls_FWD_MdPbDpSfSpTr_DM.ProgBathDay_18ID_3_allID %>%
 pull(model) %>% last()
M2 <- HMM_ls_FWD_MdPbDpSfSpTr_DM.ProgBathDay_26ID_3_allID %>%
 pull(model) %>% last()

# calculate confusion matrix
HMM_confusion_matrix(M2, M1)

Confusion Matrix and Statistics

 Reference
Prediction 1 2 3
 1 613 32 12
 2 68 1028 52
 3 23 76 876

Overall Statistics

 Accuracy : 0.9054
 95% CI : (0.8939, 0.916)
 No Information Rate : 0.4086
 P-Value [Acc > NIR] : < 2.2e-16

 Kappa : 0.8551

 Mcnemar's Test P-Value : 0.0001095

Statistics by Class:

 Class: 1 Class: 2 Class: 3
Sensitivity 0.8707 0.9049 0.9319
Specificity 0.9788 0.9270 0.9462
Pos Pred Value 0.9330 0.8955 0.8985
Neg Pred Value 0.9571 0.9338 0.9645
Prevalence 0.2532 0.4086 0.3381
Detection Rate 0.2205 0.3698 0.3151
Detection Prevalence 0.2363 0.4129 0.3507
Balanced Accuracy 0.9248 0.9160 0.9391

**S8 Fig. Winter distribution of decoded predicted states with just 2010-2012 tag programming.** (a) is the total number of steps that occurred in each cell (50 km by 50 km), which represents the total data that went into predictions for each cell (higher number of steps used indicates greater confidence in state predictions), and (b) is calculated as the state ***S***$\mathbf{}^{\mathbf{'}}$ with the highest within-cell proportion relative to the frequency of each state across all cells. Cells with < 4 steps are not plotted, and hash lines represent cells with < 16 total steps, which indicate low certainty in those state predictions.

**S9 Fig. Predicted winter distribution of decoded states with just 2010-2012 tag programming.** Behavioural decoded states: (a) 1 (green), (b) 2 (blue), and (c) 3 (red), with dark areas represent high state frequency within each cell (50 km by 50 km). Cells with < 4 steps are not plotted, and hash lines represent cells with <16 total steps.

# **References**

1. McClintock BT, London JM, Cameron MF, Boveng PL. Modelling animal movement using the Argos satellite telemetry location error ellipse. Methods Ecol Evol. 2015;6:266–77.

2. Pohle J, Langrock R, van Beest FM, Schmidt NM. Selecting the number of states in Hidden Markov Models: Pragmatic solutions illustrated using animal movement. J Agric Biol Environ Stat. 2017;22:1215–27.

3. Dupont F, Marcoux M, Hussey N, Dupont F, Auger-Méthé M. Improved order selection method for hidden Markov models: A case study with movement data. Methods Ecol Evol. 2025;16:1215–27.

4. Zucchini W, MacDonald IL, Langrock R. Hidden Markov Models for Time Series: An Introduction Using R, Second Edition. Boca Raton, FL: Chapman & Hall/CRC.; 2016. 398 p.

5. Togunov RR, Derocher AE, Lunn NJ, Auger-Méthé M. Drivers of polar bear behavior and the possible effects of prey availability on foraging strategy. Mov Ecol. 2022;10(1):1–20.
