# Supplementary figures and images for: Behavioural analysis of multi-year satellite telemetry data provides insight into narwhal (Monodon monoceros) winter prey selection in Baffin Bay
Source: PLoS One. 2025 Sep 2;20(9):e0330928. doi: 10.1371/journal.pone.0330928 (PMC12404367; doi:10.1371/journal.pone.0330928)

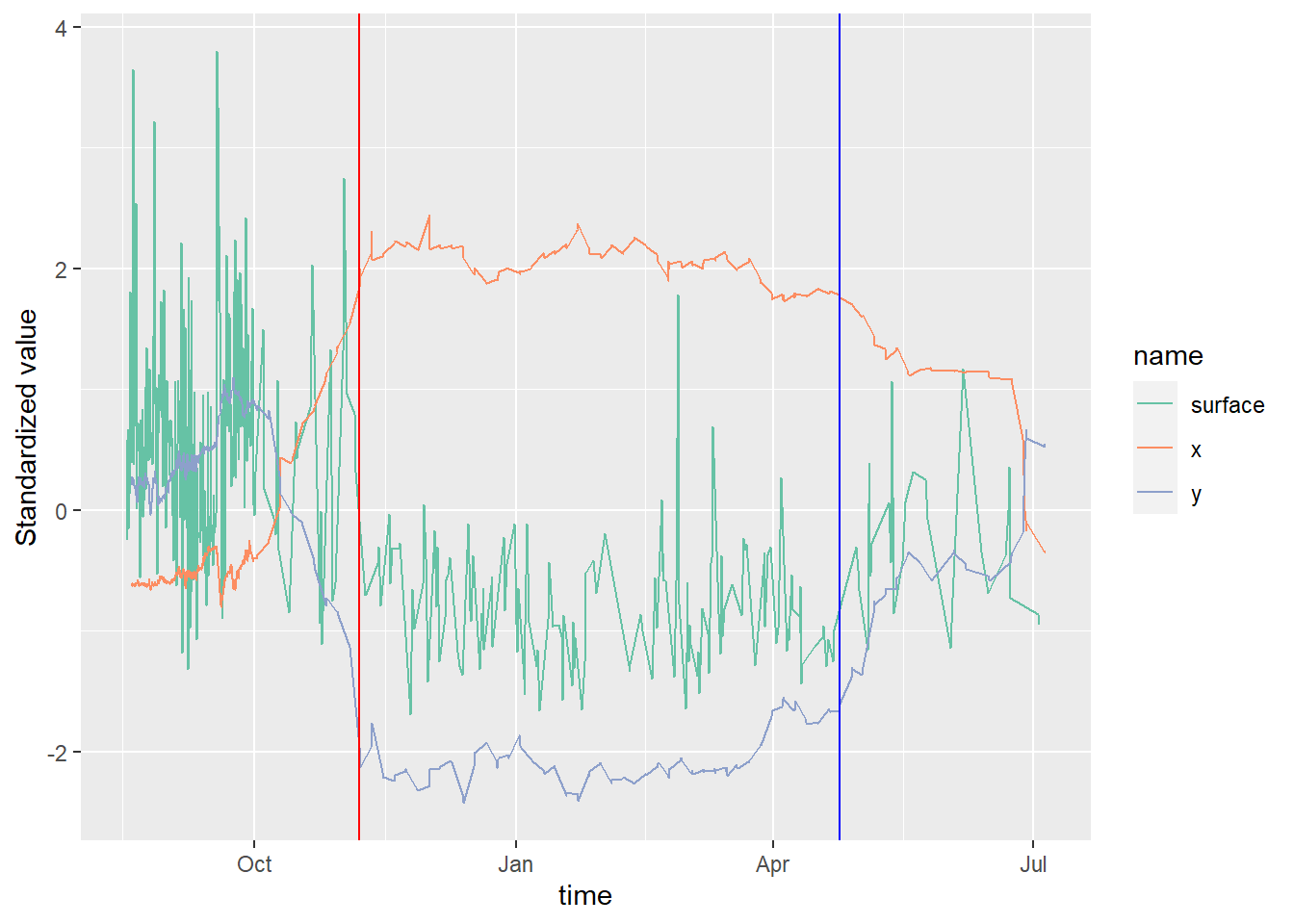

Supplement: S1 Fig — Example of definition of winter dates based on standardized value of surface time, and x and y coordinates. Beginning of winter is denoted by the vertical red line and the end of winter is denoted by the vertical blue line. (TIF) [file pone.0330928.s002.tif]

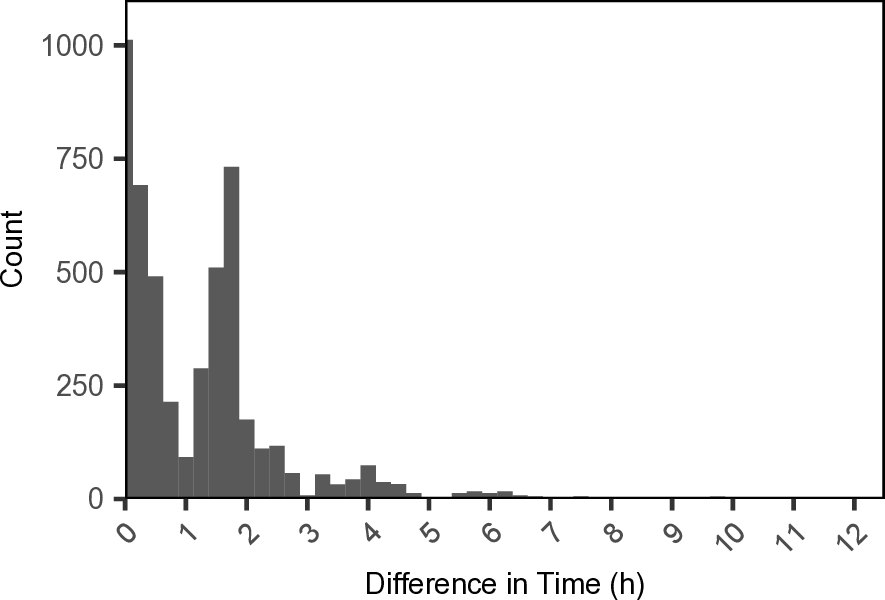

Supplement: S2 Fig — Histogram of observed data gaps of narwhal transmissions, shown as difference in time (hours). (TIF) [file pone.0330928.s003.tif]

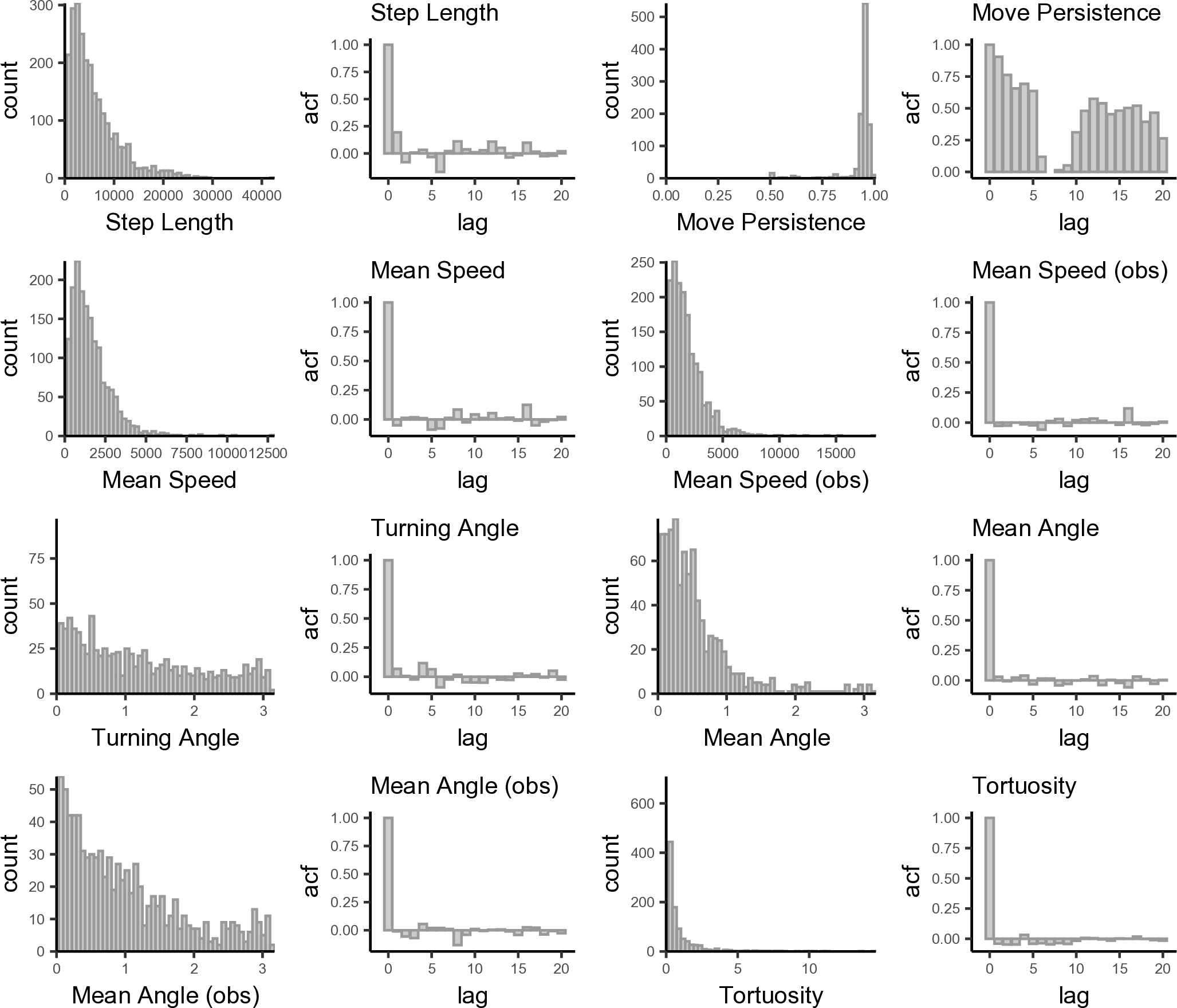

Supplement: S3 Fig — Histograms and autocorrelation functions for seven movement data streams: step length, mean speed, observed mean speed, turning angle, mean angle, observed mean angle, and move persistence (tortuosity). (TIF) [file pone.0330928.s004.tif]

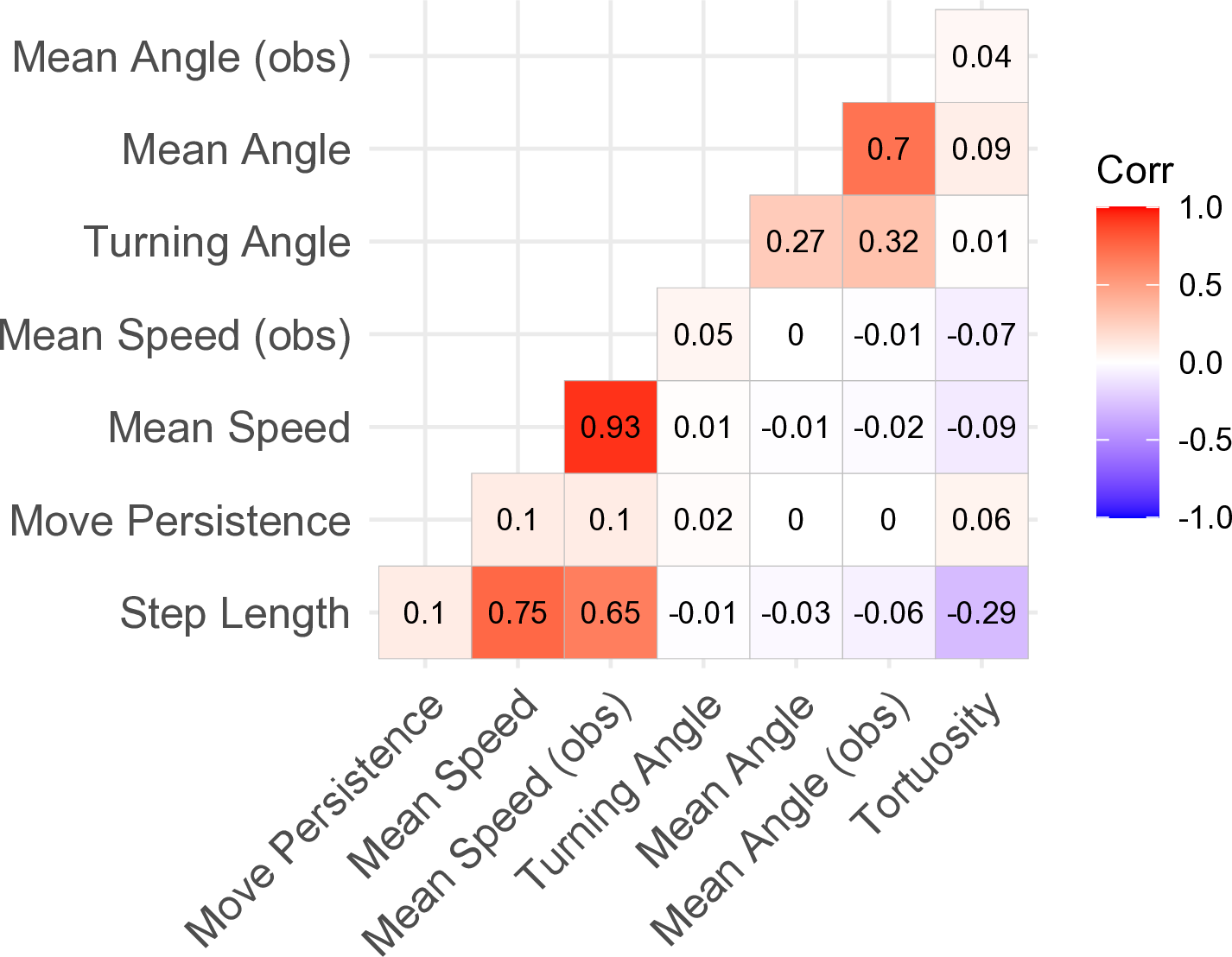

Supplement: S4 Fig — Pearson’s correlation matrix for the seven tested movement data streams: step length, mean speed, observed mean speed, turning angle, mean angle, observed mean angle, and move persistence (tortuosity). (TIF) [file pone.0330928.s005.tif]

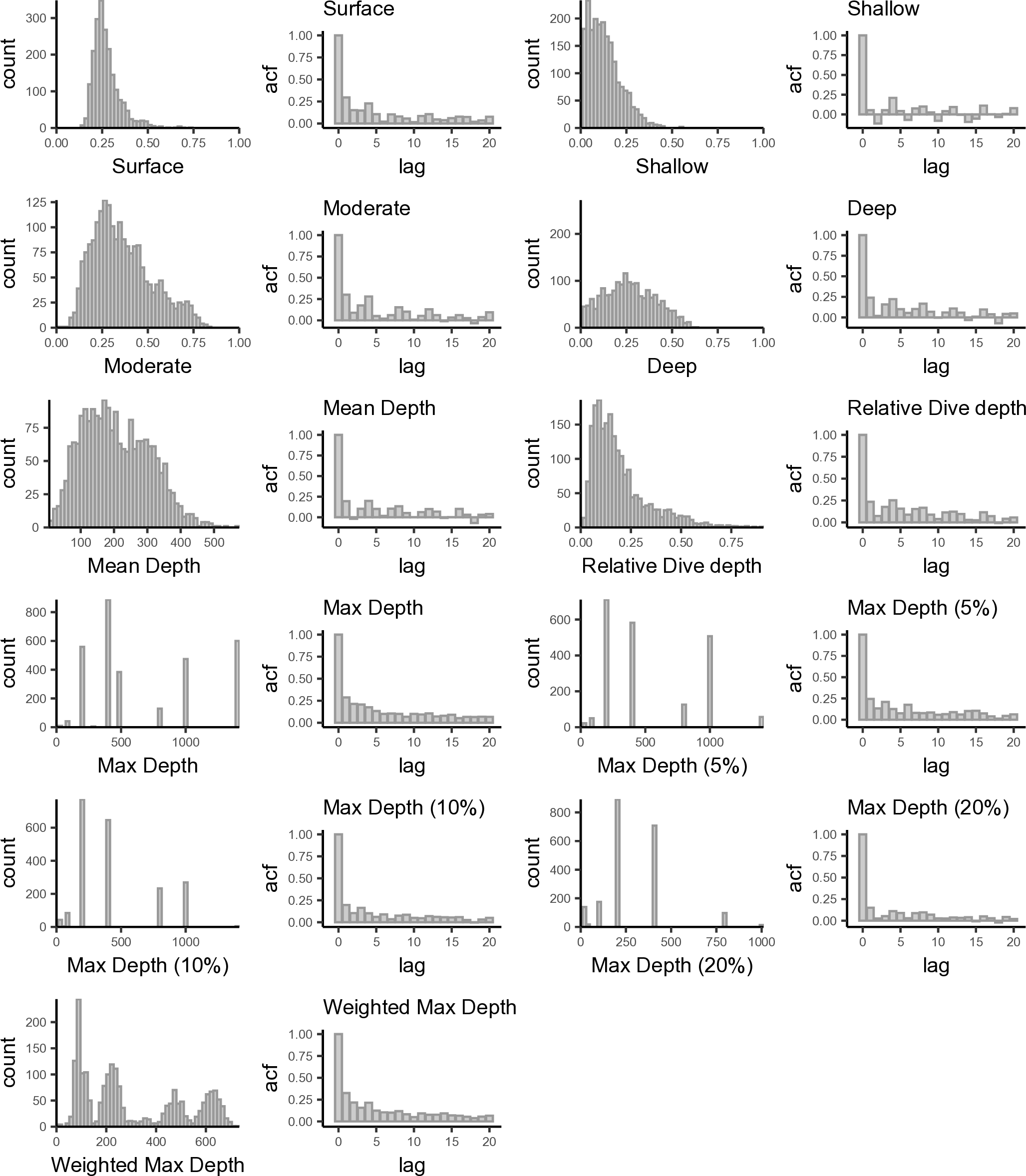

Supplement: S5 Fig — Histograms and autocorrelation functions for 11 different dive data streams: Surface, shallow, moderate, deep, mean depth, relative dive depth, max depth, max depth (5%), max depth (10%), max depth (20%), and weighted max depth. (TIF) [file pone.0330928.s006.tif]

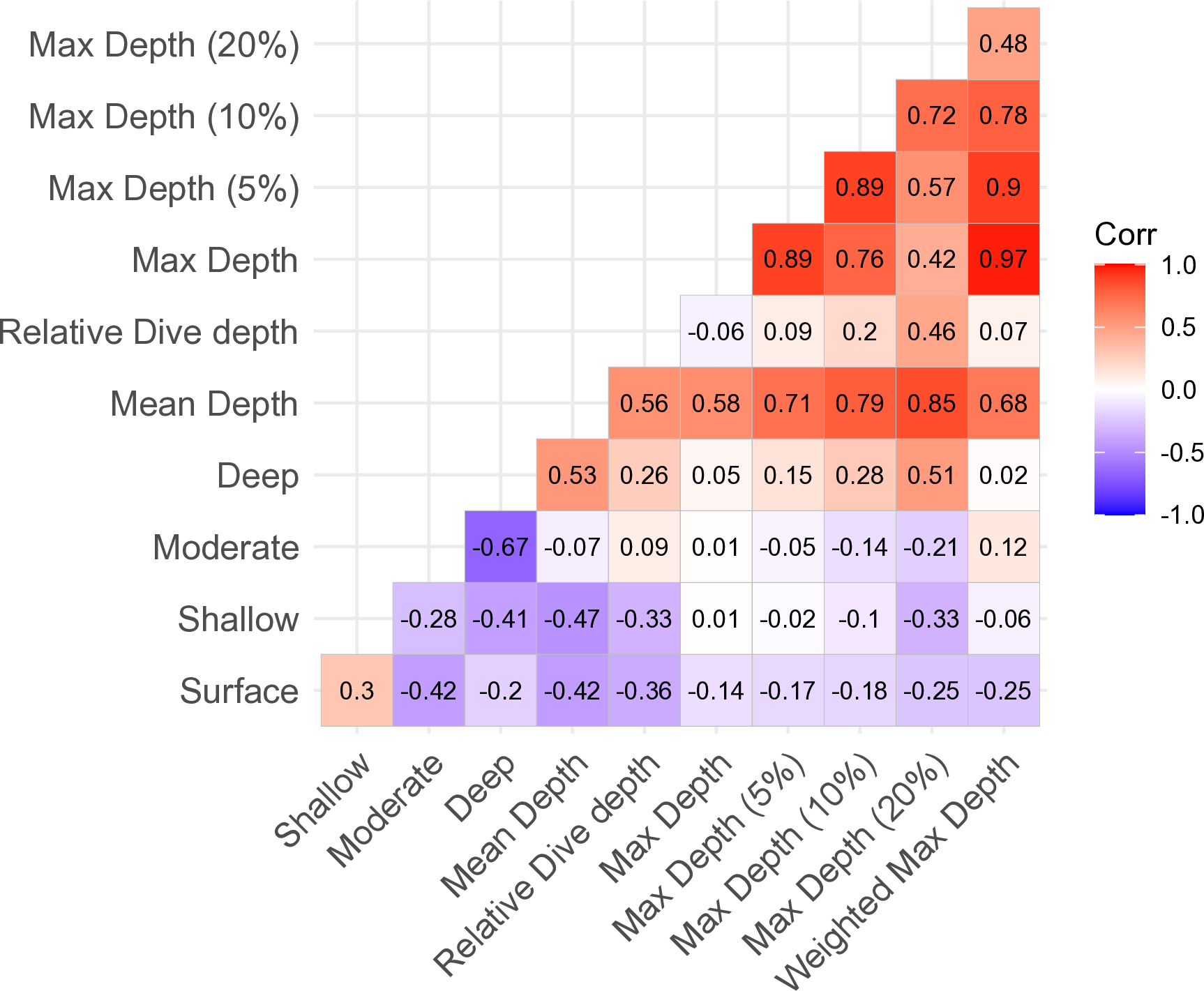

Supplement: S6 Fig — Correlation matrix for the 11 tested dive data streams: Surface, shallow, moderate, deep, mean depth, relative dive depth, max depth, max depth (5%), max depth (10%), max depth (20%), and weighted max depth. (TIF) [file pone.0330928.s007.tif]

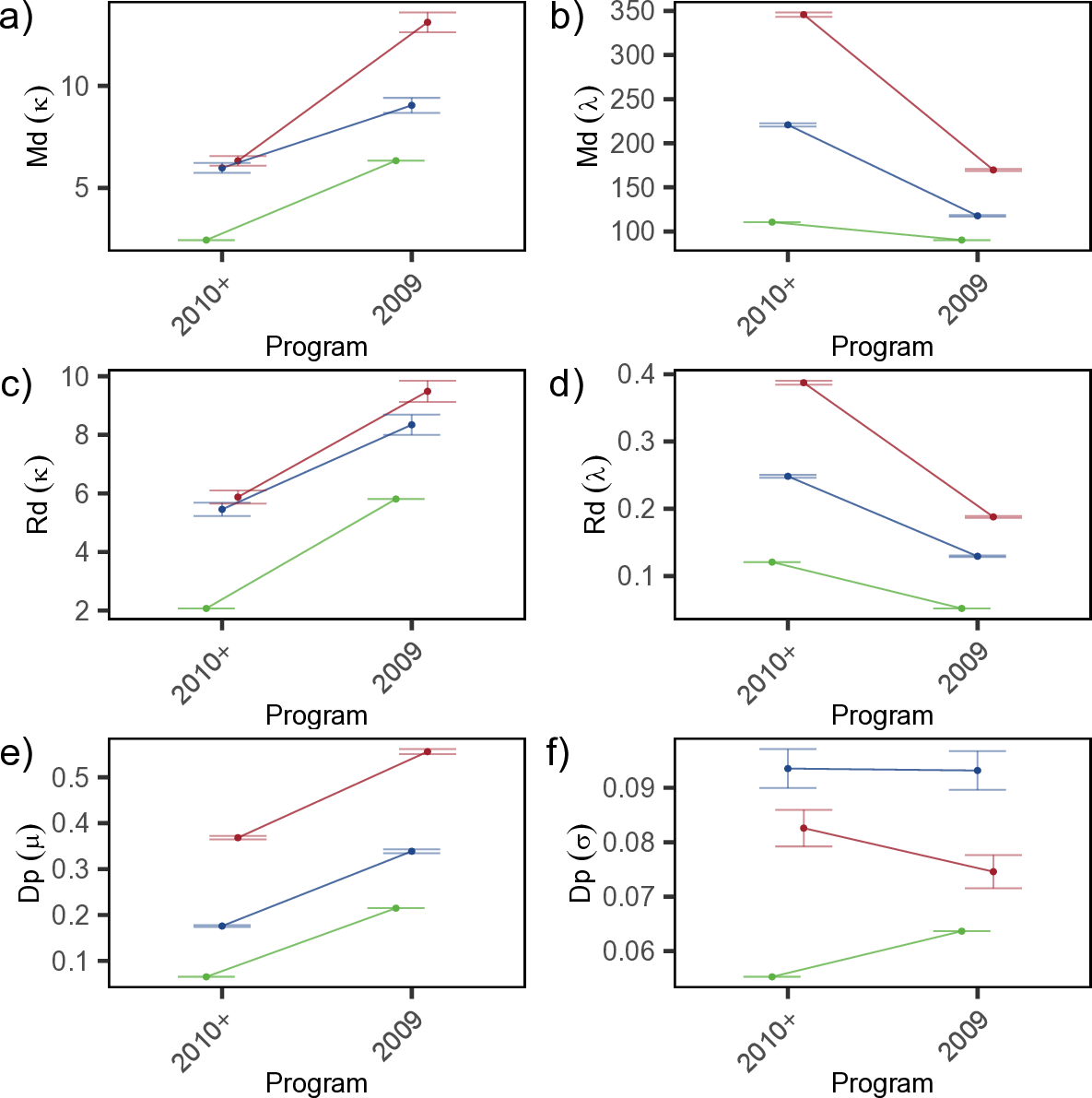

Supplement: S7 Fig — Predicted effect of tag programming on emission probabilities for the shape (κ), scale (λ), mean (μ), and standard deviation (σ) parameters of three diving data streams: mean depth (Md) and relative dive depth (Rd) and time at depth (Dp). Effects were assumed to be independent for each of the three behaviour states: state 1 (surface activity; green), state 2 (pelagic diving; blue), and state 3 (benthic diving; red). Error bars represent the estimated 95% confidence intervals of the means. (TIF) [file pone.0330928.s008.tif]

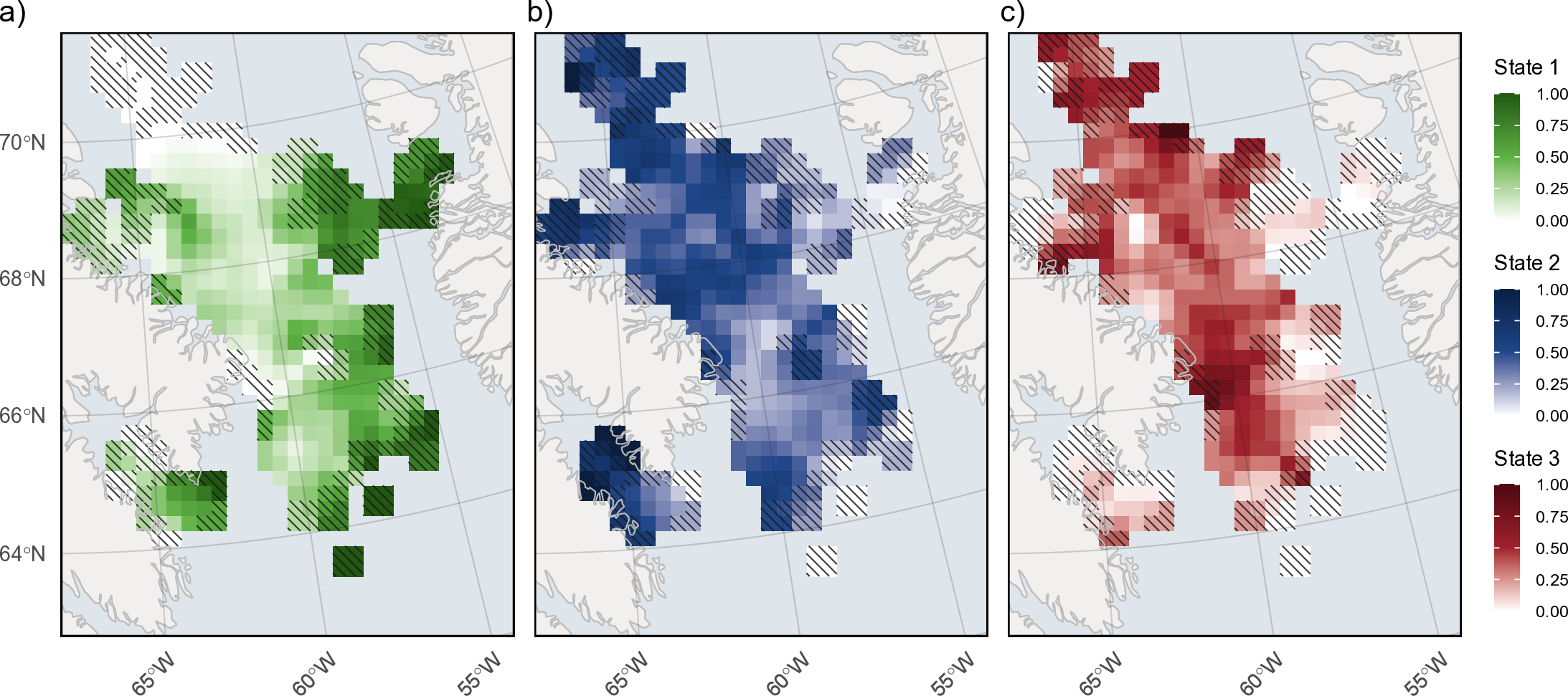

Supplement: S9 Fig — Behavioural decoded states (a) 1 (green), (b) 2 (blue), and (c) 3 (red), with dark areas representing high state frequency within each cell (50 km by 50 km). Cells with < 4 steps are not plotted, and hash lines represent cells with <16 total steps. (TIF) [file pone.0330928.s010.tif]
